# Effect of intensive care unit-specific virtual reality (ICU-VR) to improve psychological well-being and quality of life in COVID-19 ICU survivors: a study protocol for a multicentre, randomized controlled trial

**DOI:** 10.1186/s13063-021-05271-z

**Published:** 2021-05-05

**Authors:** Johan H. Vlake, Jasper Van Bommel, Evert-Jan Wils, Tim I. M. Korevaar, Merel E. Hellemons, Anna F. C. Schut, Joost A. M. Labout, Lois L. H. Schreuder, Diederik Gommers, Michel E. Van Genderen

**Affiliations:** 1 Department of Intensive Care, Erasmus MC, Dr. Molewaterplein 40, 3015 GD Rotterdam, The Netherlands; 2 Department of Intensive Care, Franciscus Gasthuis & Vlietland, Kleiweg 500, 3045 PM Rotterdam, The Netherlands; 3 Department of Internal Medicine, Academic Centre for Thyroid Diseases, Erasmus MC, Dr. Molewaterplein 40, 3015 GD Rotterdam, The Netherlands; 4 Department of Pulmonology, Erasmus MC, Dr. Molewaterplein 40, 3015 GD Rotterdam, The Netherlands; 5 Department of Intensive Care, Ikazia Hospital, Montessoriweg 1, 3083 AN Rotterdam, The Netherlands; 6 Department of Intensive Care, Maasstad Hospital, Maasstadweg 21, 3079 DZ Rotterdam, The Netherlands

**Keywords:** Coronavirus disease 2019 (COVID-19), SARS-CoV-2, Post-intensive care syndrome (PICS), Post-traumatic stress disorder (PTSD), Anxiety, Depression, Health-related quality of life (HRQoL), Virtual reality, Randomized controlled trial

## Abstract

**Background:**

The SARS-CoV-2 outbreak has resulted in a tremendous increase in hospital and intensive care unit (ICU) admissions all over the world. Patients with severe coronavirus disease 2019 (COVID-19) warranting ICU treatment usually have prolonged mechanical ventilation and are expected to be prone to develop psychological impairments, such as post-traumatic stress disorder (PTSD), anxiety and depression, which negatively impact quality of life. To date, no effective treatment strategy is available. In the current trial, we aim to assess the effect of an ICU-specific virtual reality (ICU-VR) intervention on psychological well-being and quality of life after COVID-19 ICU treatment.

**Methods:**

In this multicentre, randomized controlled trial, we aim to examine whether COVID-19-specific ICU-VR, offered 3 months after hospital discharge, improves psychological well-being and quality of life. Secondary objectives are, firstly, to examine the intra-group changes in psychological well-being and quality of life and the inter-group differences in psychological well-being and quality of life during follow-up, up to 12 months after hospital discharge, and secondly, to examine patients’ satisfaction with and rating of ICU care and aftercare and patients’ perspectives on ICU-VR. Eighty adult patients treated for COVID-19 in the mixed-surgical ICUs of four hospitals in Rotterdam, the Netherlands, will be included and randomized (1:1) to either early or late ICU-VR between June 29 and December 31, 2020. Patients randomized to early ICU-VR will receive the ICU-VR intervention during an outpatient clinic visit 3 months after hospital discharge, whereas patients randomized to late ICU-VR will receive ICU-VR 6 months after hospital discharge. Primary outcomes of this study are psychological well-being, assessed using the Impact of Event Scale–Revised (IES-R) and the Hospital Anxiety and Depression Scale (HADS), and quality of life, assessed using the European Quality of Life 5 Dimensions (EQ-5D) and RAND-36 questionnaires, up to 6 months after hospital discharge.

**Discussion:**

Currently, an effective treatment for psychological sequelae after ICU treatment for specific illnesses is unavailable. Results from this study will provide insight whether virtual reality is a modality that can be used in ICU aftercare to improve psychological well-being and quality of life, or satisfaction, after ICU treatment for specific illnesses such as COVID-19.

**Trial registration:**

This trial has been retrospectively registered on the Netherlands Trial Register on August 14, 2020 (NL8835).

**Supplementary Information:**

The online version contains supplementary material available at 10.1186/s13063-021-05271-z.

## Administrative information

Note: The numbers in curly brackets in this protocol refer to SPIRIT checklist item numbers. The order of the items has been modified to group similar items (see http://www.equator-network.org/reporting-guidelines/spirit-2013-statement-defining-standard-protocol-items-for-clinical-trials/).

**Table:** 

Title {1}	Effect of intensive care unit-specific virtual reality (ICU-VR) to improve psychological distress and quality of life in COVID-19 ICU survivors: study protocol for a multicentre, randomized controlled trial.
Trial registration {2a and 2b}.	Registry: www.trialregister.nl Identifier: NL8835 (http://www.trialregister.nl/trial/8835) Date registered: August 14, 2020
Protocol version {3}	Version 4.0, July 2020
Funding {4}	Sitchting Theia (foundation): Subsidy to develop the COVID-19 ICU-VR intervention. Stichting SGS (foundation): Subsidy to develop the COVID-19 ICU-VR intervention. BeterKeten (foundation): Subsidy for the PhD-trajectory of Johan H. Vlake
Author details {5a}	1. **Johan H. Vlake, BSc** J.Vlake@erasmusmc.nl Department of Intensive Care Erasmus Medical Centre Dr. Molewaterplein 40, 3015 GD Rotterdam, the Netherlands Department of Intensive Care, Franciscus Gasthuis & Vlietland Kleiweg 500, 3045 PM Rotterdam, the Netherlands 2. **Jasper van Bommel, MD, PhD** J.VanBommel@erasmusmc.nl Department of Intensive Care Erasmus Medical Centre Dr. Molewaterplein 40, 3015 GD Rotterdam, the Netherlands 3. **Evert-Jan Wils, MD, PhD** E.Wils@franciscus.nl Department of Intensive Care Franciscus Gasthuis & Vlietland Kleiweg 500, 3045 PM Rotterdam, the Netherlands 4. **Tim I.M. Korevaar, MD, PhD** T.Korevaar@erasmusmc.nl Department of Internal Medicine Academic Centre for Thyroid Diseases Erasmus Medical Centre Dr. Molewaterplein 40, 3015 GD Rotterdam, the Netherlands 5. **Merel E. Hellemons, MD, PhD** M.Hellemons@erasmusmc.nl Department of Pulmonology Erasmus Medical Centre Dr. Molewaterplein 40, 3015 GD Rotterdam, the Netherlands 6. **Anna F.C. Schut, MD, PhD** A.Schut@ikazia.nl Department of Intensive Care Ikazia Hospital Montessoriweg 1, 3083 AN Rotterdam, the Netherlands 7. **Joost A.M. Labout, MD, PhD** LaboutJ@maasstadziekenhuis.nl Department of Intensive Care Maasstad Hospital Maasstadweg 21, 3079 DZ Rotterdam, the Netherlands 8. **Lois L.H. Schreuder, BSc** L.L.H.Schreuder@erasmusmc.nl Department of Intensive Care Erasmus Medical Centre Dr. Molewaterplein 40, 3015 GD Rotterdam, the Netherlands 9. **Diederik Gommers, MD, PhD** D.Gommers@erasmusmc.nl Department of Intensive Care Erasmus Medical Centre Dr. Molewaterplein 40, 3015 GD Rotterdam, the Netherlands 10. **Michel E. Van Genderen, MD, PhD** M.VanGenderen@erasmusmc.nl Department of Intensive Care Erasmus Medical Centre Dr. Molewaterplein 40, 3015 GD Rotterdam, the Netherlands
Name and contact information for the trial sponsor {5b}	**Erasmus Medical Centre** Doctor Molewaterplein 40 3015 GD Rotterdam The Netherlands
Role of sponsor {5c}	Neither the sponsor, nor the funding sources, had any role in the study design; collection, management, analysis, and interpretation of data; writing the manuscript of the protocol, or the decision to submit the protocol for publication. They will not have any authority over any of these activities.

## Introduction

### Background and rationale {6a}

The SARS-CoV-2 pandemic in December 2019 has resulted in a tremendous increase in hospital and intensive care unit (ICU) admissions [1–4]. While initial reports from China indicated that approximately 5% of patients with SARS-CoV-2 were admitted to the ICU, reports from Italy suggest that this number may be as high as 16%, leading to stressed ICU capacity [5, 6].

Critically ill patients with severe pulmonary disease treated in the ICU are known to develop long-term impairments [7–10]. These impairments consist of psychological, physical and cognitive impairments and are collectively referred to as the post-intensive care syndrome (PICS) [11–14]. The psychological component of PICS consists of post-traumatic stress disorder (PTSD), anxiety and depression, is the most important determinant of a decreased health-related quality of life and negatively impacts a patients’ ability to rehabilitate [8, 15–17].

Prevention and treatment of PICS is a major objective to achieve a sustained improvement in the quality of ICU care in the decades to come. Despite growing awareness, several interventions aiming to improve psychological well-being have yielded unsatisfactory and ambiguous results [18–22]. Psychological sequelae after ICU exposure are hypothesized to reflect a combination of sensory overload and delusional memories [23–25]. Veracious reconstruction of memories to fill in memory gaps and reframe delusional memories may reduce these psychological symptoms [26].

Virtual reality (VR) is a relatively new technique that has been proven to be effective for treating several psychological impairments, including PTSD and anxiety disorders [27–30]. VR has three major advances: first, it represents a means of addressing the limitations of imaginal exposure and overcomes a significant hurdle of imaginal exposure, the inability to engage in sufficient detail, and affective magnitude to recreate the traumatic event; second, it is an appropriate tool for patient information delivery; and third, using VR, one can truthfully reconstruct phases of ICU treatment to replace and adjust possible delusional memories, the largest contributor to psychological distress [26, 31].

ICU-specific VR (ICU-VR) is safe and immersive and improves psychological well-being and mental quality of life in sepsis survivors using a median of 2 VR sessions [32, 33]. It is however unknown whether these findings can be extrapolated to a broader ICU population, and if such a modality can be structurally implemented in ICU aftercare, such as in post-ICU follow clinics, where patients are first invited at 3 months after hospital discharge [34]. In the current trial, we therefore aim to extend our previous findings and examine the effect of ICU-VR 3 months after hospital discharge on psychological well-being and quality of life in patients treated for COVID-19.

## Objectives {7}

The primary objective is to examine whether COVID-19 specific ICU-VR, 3 months after hospital discharge, improves psychological well-being and quality of life. Secondary objectives are, firstly, to examine the intra-group changes in psychological well-being and quality of life and the inter-group differences in psychological well-being and quality of life during follow-up, up to 12 months after hospital discharge, and secondly to examine patients’ satisfaction with and rating of ICU care and aftercare and patients’ perspectives on ICU-VR.

## Trial design {8}

A multicentre, open-label, randomized controlled, superiority, crossover trial.

## Methods: participants, interventions and outcomes

### Study setting {9}

This multicentre, randomized controlled, open-label trial will be conducted in the mixed-medical ICUs of four hospitals in Rotterdam, the Netherlands: one university hospital providing tertiary care (Erasmus MC) and three teaching hospitals providing secondary care (Franciscus Gasthuis & Vlietland Hospital, Ikazia Hospital and Maasstad Hospital).

### Eligibility criteria {10}

We aim to include patients older than 18 years of age with COVID-19, determined by a positive SARS-CoV-2 PCR, necessitating ICU care. Patients discharged from the hospital between March 29 and September 30, 2020, and who are able to understand the Dutch language are eligible for inclusion. Patients will be excluded when they suffer from active, established psychiatric disease, for instance personality disorders or schizophrenia; are admitted with a history or a primary neurological impairment necessitating ICU treatment; have no formal home address; or are enrolled in other interventional studies that could confound the primary endpoint.

### Who will take informed consent? {26a}

Patients admitted for COVID-19 to the ICU of the participating hospitals will be invited to a post-COVID-19 outpatient clinic 3 months after hospital discharge as part of the regional standard of care. All patients will receive an information letter and will be contacted by telephone by one of the members of the research team to discuss participation 1 month prior to this visit. During the outpatient clinic visit, informed consent will be obtained by the principal investigator of the study site, or by one of the other members of the research team if the principal investigator is unavailable.

### Additional consent provisions for collection and use of participant data and biological specimens {26b}

Not applicable, no additional participant data or biological specimens were obtained.

## Interventions

### Explanation for the choice of comparators {6b}

In this study, patients in both randomization groups (i.e. the early and late ICU-VR group) will receive the intervention, either 3 or 6 months after hospital discharge. As such, no comparator is used.

### Intervention description {11a}

Both groups will receive the ICU-VR intervention during the study period, 3 (early) and 6 (late) months after hospital discharge. ICU-VR (duration: ±14 min) comprises several modules explaining the aspects of ICU treatment that are known to be the most frightening [24, 35]. The content was previously determined by a multidisciplinary team and has been demonstrated to be safe [32, 33]. For COVID-19 ICU survivors, we adapted the latter module by adding additional COVID-19 specific aspects of ICU treatment (i.e. mechanical ventilation in prone position, tracheostomy and isolation measures). In addition, information regarding SARS-CoV-2 and COVID-19 was added, and the voice-over was changed accordingly. Real ICU nurses and physicians were used to re-enact a typical day/treatment for a mock patient undergoing ICU treatment for COVID-19.

**Fig. 1:**
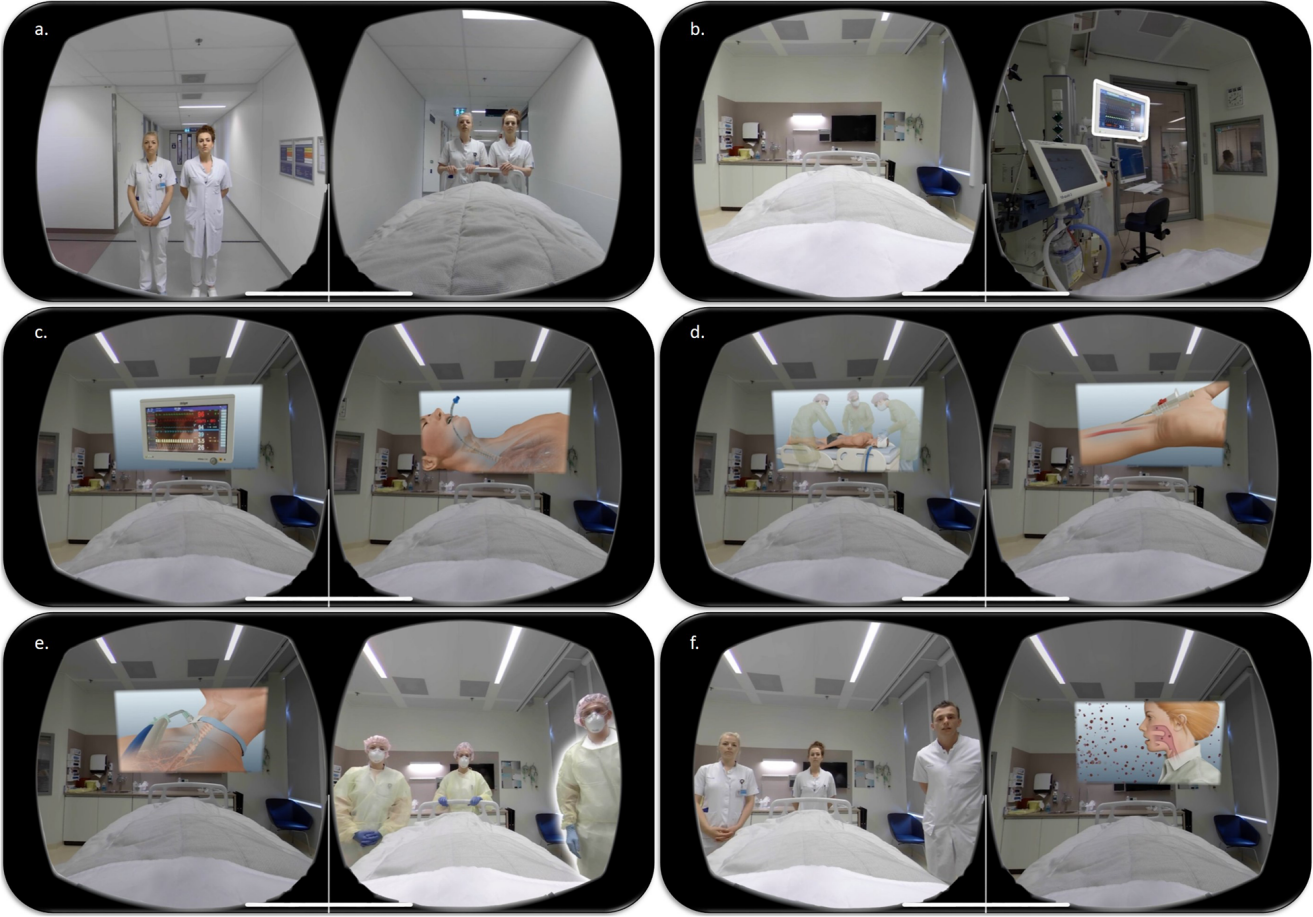
Impression of the COVID-19 ICU-VR intervention. Screenshots of the COVID-19 ICU-VR intervention; the ICU physician and nurse welcome the patient while standing in front of the ICU (**a**, left), where the patients are virtually installed in an ICU bed (**a**, right). After the ICU physician and nurse have brought the patient to the ICU room while walking over the ICU, the patient will be placed in the ICU room (**b**, left), where the patient receives an explanation about the surveillance monitor (**b**, right; **c**, left), intubation (**c**, right), prone positioning (**d**, left), drips and infuses (**d**, right), tracheotomy (**e**, left), isolation and personal protection measures (**e**, right), the treatment team (**f**, left) and SARS-CoV-2/COVID-19 (**f**, right). A full overview of the content of the COVID-19 ICU-VR intervention can be found in Additional file 1

ICU-VR is hospital-specific, to expose patients to the actual environment where they were treated. As such, each hospital has its unique ICU-VR. An overview of the ICU-VR intervention of the Erasmus MC is depicted in Fig. 1, and the film script can be found in Additional file 1 (translated from Dutch to English). The intervention will be watched via head-mounted display (HMD-)VR glasses (Oculus Go, Irvine, CA, www.oculus.com/go), and patients will be allowed to move their head freely as to experience all aspects of the virtual environment.

### Criteria for discontinuing or modifying allocated interventions {11b}

Not applicable. Patients undergo ICU-VR once in the hospital. As such, discontinuation or modification of the allocated intervention is not possible.

### Strategies to improve adherence to interventions {11c}

Not applicable. Patients undergo ICU-VR once in the hospital. No further adherence is needed.

### Relevant concomitant care permitted or prohibited during the trial {11d}

Participants randomized to the late ICU-VR group require an additional visit to an outpatient clinic to receive ICU-VR. No additional alterations to usual care pathways, including the use of any medication, are required for participants in this study.

### Provisions for post-trial care {30}

Due to the absence of anticipated harm of participation in this study, the accredited medical ethics committee has granted dispensation from the statutory obligation to provide insurance to participants through injury or death caused by the study. The sponsor has a liability insurance which is in accordance with national legislation. Participants will not be compensated for their participation in the study.

### Outcomes {12}

The primary outcome is the effect of early ICU-VR on psychological well-being, expressed as symptoms of PTSD, anxiety and depression and quality of life up to 6 months after hospital discharge. Secondary outcomes are, firstly, the intra-group changes in psychological well-being and quality of life and the inter-group differences in psychological well-being and quality of life during follow-up up to 12 months after hospital discharge, and secondly, patients’ satisfaction with and rating of ICU care and aftercare and patients’ perspectives on ICU-VR.

### Participant timeline {13}

**Fig. 2:**
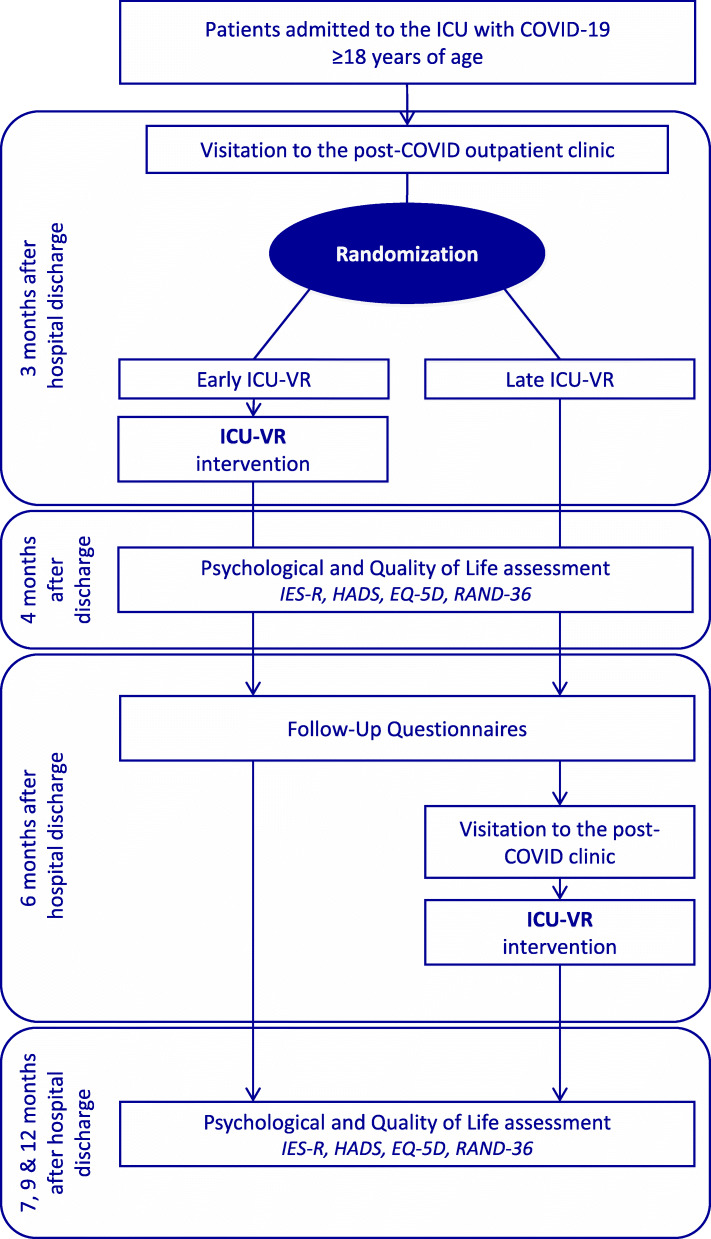
Flow diagram of the study. Abbreviations: COVID-19, coronavirus disease 2019; EQ-5D, European Quality of life 5 dimension questionnaire; HADS, Hospital Anxiety and Depression Scale; ICU, intensive care unit; ICU-VR, intensive care unit-specific virtual reality; IES-R, Impact of Event Scale–Revised. Eligible patients will be invited to a post-COVID outpatient clinic and randomized across the early and late ICU-VR group. The early group will receive the ICU-VR intervention during the same visit, while the late group receives ICU-VR during a second outpatient clinic visitation 6 months after hospital discharge. Psychological well-being and quality of life will be assessed 3, 4, 6, 7 and 12 months after hospital discharge using follow-up questionnaires

Figure 2 depicts participants’ recruitment and randomization, the study procedures and the outcomes of the study. Patients admitted for COVID-19 to the ICU of the participating hospitals will be invited to a post-COVID-19 outpatient clinic 3 months after hospital discharge as part of the regional standard of care. All patients will receive an information letter and will be contacted by telephone to discuss participation 1 month prior to this visit. During the outpatient clinic visit, informed consent will be obtained and patients will be randomized. Patients randomized to the early ICU-VR group will receive ICU-VR during the same visit, whereas patients randomized to the late ICU-VR group will receive ICU-VR during a second outpatient clinic visit 3 months later (i.e. 6 months after hospital discharge). Questionnaires will be sent at 3 months (prior to the first outpatient clinic visit), 4 months, 6 months (prior to the second outpatient clinic visit for patients in the late intervention group), 7 months and 12 months after hospital discharge.

### Sample size {14}

In the previously conducted pilot study examining the feasibility, safety and clinical relevance of ICU-VR in sepsis survivors, we identified an effect size Cohen’s *d* of 0.77 [33]. Due to the non-normality of PTSD scores after 6 months, these calculations could represent an overestimation of the sample size. We consider the effect estimates for the ICU-VR module to be similar to the intervention group, as was tested in our pilot study. A G*Power analysis revealed that 80 patients have to be included in the study to detect such an effect size, using a two-sided alpha of 0.05, a power of 0.80, a 1:1 randomization and an expected loss to follow-up of 20% based on our pilot study. We will include patients from June 2020 to December 2020. When the minimum required number of patients is reached prior to December 2020, inclusion will be continued until December 2020. When the minimum required number of patients is not reached in December 2020, inclusion will be continued until the minimum required sample of 80 patients is reached.

### Recruitment {15}

Patients will receive information 1 month prior to the post-COVID outpatient clinic visit and will be recruited during the post-COVID outpatient clinic visit, after being given the opportunity to ask questions regarding participation.

## Assignment of interventions: allocation

### Sequence generation {16a}

Patients will be randomly assigned to either early ICU-VR, receiving the intervention 3 months after discharge, of late ICU-VR, receiving the intervention 6 months after hospital discharge. Randomization will be a 1:1 allocation as per computer-generated randomization schedule stratified by site using permuted blocks of random sizes. The block sizes will not be disclosed, to ensure concealment.

### Concealment mechanism {16b}

Patients will be randomized using the Castor Electronic Data Capture system (Castor EDC, https://www.castoredc.com, Amsterdam, the Netherlands), an online data capture system including a central randomization service.

### Implementation {16c}

After informed consent has been obtained, patients will be randomized by the principal investigator of each study site using Castor EDC during the post-COVID outpatient clinic at 3 months after hospital discharge. After randomization, Castor EDC reveals the allocation of the patient, where after the patient will receive ICU-VR if allocated to early ICU-VR, or will be invited to a second outpatient clinic 6 months after hospital discharge if allocated to late ICU-VR.

## Assignment of interventions: blinding

### Who will be blinded {17a}

Due to the nature of the intervention, neither the patients nor the investigator can be blinded to the randomization allocation. The allocation data within the dataset will be presented as either ‘0’ or ‘1’, without the corresponding randomization allocation. Thereby, the investigator performing the analysis will be blinded to the randomization allocation.

### Procedure for unblinding if needed {17b}

Not applicable, neither participants nor investigators are blinded to the randomization allocation.

## Data collection and management

### Plans for assessment and collection of outcomes {18a}

Psychological well-being will be expressed as the presence and severity of PTSD, anxiety and depression, which will be assessed using the Impact of Event Scale-Revised (IES-R) and Hospital Anxiety and Depression Scale (HADS) [36, 37]. Quality of life will be assessed using the European Quality of Life 5-dimensions (EQ-5D) and RAND-36 questionnaires [38, 39]. Patients’ satisfaction with and rating of ICU care and aftercare and patients’ perspectives on ICU-VR will be assessed using a novel questionnaire.

The IES-R comprises 22 items, assesses subjective distress caused by a traumatic event and has been previously validated in ICU survivors [40]. The IES-R yields a total score (ranging from 0 to 88, with higher scores indicating more severe symptoms), and subscale scores can be calculated for symptoms of intrusion, avoidance and hyperarousal. An IES-R sum score ≥ 24 will be considered as PTSD [41]. The HADS comprises 14 items and is commonly used to determine the levels of anxiety and depression that a patient is experiencing. Seven of the items relate to anxiety and seven relate to depression. A sum score above 8 on either the depression or anxiety subscale will be classified as depression and anxiety, respectively [36].

The EQ-5D measures quality of life in five dimensions (mobility, self-care, usual activities, pain/discomfort and anxiety/depression), from which the weight of a health state can be computed, the EQ-5D utility score, ranging from − 0.446 (worst quality of life) to 1.000 (best quality of life) [42]. Additionally, patients score their current subjective health on a visual analogue scale (EQ-VAS), ranging from 0 (worst health imaginable) to 100 (best health imaginable). The RAND-36 is a 36-item, patient-reported survey of patient health and quality of life. The RAND-36 consists of eight scaled scores, which are the weighted sums of the questions in their section. Each scale is directly transformed to a scale ranging from 0 to 100 on the assumption that each question carries an equal weight. The eight sections are vitality, physical functioning, bodily pain, general health perception, physical role functioning, emotional role functioning, social role functioning and mental health [39].

Patients’ satisfaction with and rating of ICU care and aftercare was based on the Patient Satisfaction Questionnaire and Family Satisfaction with ICU Care tools, altered to the needs of this study [43–45]. Additional novel items were added to evaluate patients’ perspectives on the ICU-VR intervention.

This questionnaire comprised 21 items, categorized in four sections: (1) satisfaction with and rating of ICU care and ICU aftercare and the added value of ICU-VR; (2) overall perspectives on the ICU-VR intervention; (3) perspectives on the content of ICU-VR; and (4) perspective on the effect of ICU-VR. Questions regarding ICU-VR were only answered by patients who had received the intervention, questions regarding satisfaction and rating by all patients. Psychological well-being and health-related quality of life will be assessed at 3, 4, 6, 7 and 12 months after hospital discharge. Satisfaction with and rating of ICU care and aftercare and perspectives on ICU-VR were administered by telephone during the study period after the first outpatient clinic visit. Baseline demographics and treatment-related characteristics, including, but not limited to, age, gender, race, pre-existing comorbidities, hospital and ICU length of stays, mechanical ventilation-related data (duration, prone positioning, highest PaO_2_/FiO_2_ ratio, occurrence of delirium, sedation, illness severity scores (APACHE IV, SAPS II)), mortality and medication, will be retrieved from electronic patient records.

### Plans to promote participant retention and complete follow-up {18b}

Patients will be contacted by telephone when a questionnaire needs to be filled in to improve follow-up completion. If a participant decides to discontinue their participation, the reason for the discontinuation will be recorded.

### Data management {19}

Data will be uploaded, stored and maintained on the electronic data capture system of Castor EDC. The study team will be responsible for all data entry and quality control activities. The data will be checked by at least two persons from the study team and will be stored for at least 15 years on either the Castor EDC server or as a hardcopy in the ICUs of the participating hospitals. Questionnaires will preferably be sent digitally using either Castor EDC or Gezondheidsmeter PGO+ (Gezondheidsmeter, Amsterdam, the Netherlands). The latter is an online CE-certified system to digitally monitor patients and is used by all the hospitals participating in the COVID-19 aftercare programme. Patients who are unable to fill out the questionnaires online will receive hardcopy questionnaires by postal mail.

### Confidentiality {27}

To maintain anonymity, collected data will be coded with a code number, and this number will be the only reference to patient identification throughout the study. The principal investigator is the only one in possession of the translation key, making it impossible to link data to the patient. Informed-consent forms will be kept in a locked cabinet in a limited-access room at the Erasmus MC. Patient data will be stored on each local hospital’s secured server, and only the local researcher will have access to the data files, which will be stored independent of the allocation data. Data will be archived for 15 years. The handling of personal data complies with the Dutch law.

### Plans for collection, laboratory evaluation and storage of biological specimens for genetic or molecular analysis in this trial/future use {33}

Not applicable, no biological specimens were sampled.

## Statistical methods

### Statistical methods for primary and secondary outcomes {20a}

Baseline demographics and treatment-related characteristics will be quantified using descriptive statistics. Continuous variables will be presented as medians (95% range). Categorical variables will be presented as absolute numbers and relative frequencies.

Differences between study groups in continuous variables, such as the IES-R sum score, the HADS anxiety and depression scores, the RAND-36 subscales and the EQ-5D utility score, at several follow-up time points will be analysed using a mixed effect linear regression model with a random intercept for each study site. Patients will also be categorized based on clinically meaningful cut-offs for the IES-R sum score and the HADS anxiety and depression scores. An IES-R sum score ≥ 24 will be considered as clinically relevant PTSD; a HADS anxiety or depression score > 8 will be considered as clinically relevant anxiety or clinically relevant depression, respectively [36, 41, 46]. Differences in categorical variables between study groups at several follow-up time points will be analysed using a mixed effect logistic regression model with a random effect for each site. Differences in continuous or categorical variables throughout follow-up will be analysed using a mixed effect linear or logistic regression model with an interaction variable of time*randomization and a random intercept and/or slope for each individual and each study site as appropriate.

To determine whether there are time-specific windows of opportunity (3 or 6 months after hospital discharge) for the effect of ICU-VR, we will analyse psychological impairments and quality of life 1 month (4 and 7 months after hospital discharge in the early and late intervention groups, respectively), 3 months (6 and 9 months after hospital discharge in the early and late intervention groups, respectively) and 6 months after receiving ICU-VR (nine and 12 months after hospital discharge in the early and late intervention groups, respectively) using mixed effect linear/logistic regression models with a random intercept for each site and an interaction variable time*randomization.

All data will be gathered using Castor EDC. All analyses will be performed using SPSS (version 24.0; SPSS Inc., Chicago, IL) and R for Statistics (R Foundation for Statistical Computing, Vienna, Austria, 2015). A *P* value ≤ 0.05 will be considered statistically significant.

### Interim analyses {21b}

Not applicable, no interim analyses are needed to be performed.

### Methods for additional analyses (e.g. subgroup analyses) {20b}

Not applicable, no subgroup analyses will be performed.

### Methods in analysis to handle protocol non-adherence and any statistical methods to handle missing data {20c}

Missing data due to follow-up will be dealt with using multiple imputation according to the Markov-chain Monte Carlo method in cases of missing data (completely) at random, and we will additionally perform a sensitivity analysis using the Last Observation Carried Forward method.

### Plans to give access to the full protocol, participant level-data and statistical code {31c}

The full protocol, participant-level dataset and statistical code will be available from the corresponding author on reasonable request.

## Oversight and monitoring

### Composition of the coordinating centre and trial steering committee {5d}

The principal investigators of each study site are responsible for the conduct of the study and day-to-day operations. The trial steering committee has designed the study and consulted a psychologist and psychiatrist for this purpose. One member of the trial steering committee has weekly meetings with the principal investigators of each study site. The trial steering committee is responsible for the continuation of the study, ensuring that the study protocol is followed at each study site, for data collection and for amendments of the study protocol, if necessary. All data will be validated by two members of the trial steering committee. All analyses will be performed by a member of the trial steering committee and will be checked by the statistician of the trial steering committee. The trial steering committee will meet once a month to discuss study progress.

### Composition of the data monitoring committee, its role and reporting structure {21a}

This study has negligible risks for patients. ICU-VR in a non-COVID-19 setting has previously been tested safe [32, 33]. Therefore, no data monitoring committee is needed.

### Adverse event reporting and harms {22}

Adverse events (AEs) reported by the participants or observed by the investigators will be recorded. Serious adverse events (SAEs) will be reported the principal investigator without undue delay after obtaining knowledge of the events. The principal investigator will report the SAE to the medical ethics committee of the Erasmus MC within 7 days of first knowledge for SAEs that result in death or are life-threatening, followed by a period of 8 days to complete the initial preliminary report. All other SAEs will be reported within a period of maximum 15 days after the investigator has first knowledge of the SAE. All AEs will be followed until they have abated, or until a stable situation has been reached. Depending on the event, follow up may require additional tests or medical procedures as indicated and/or referral to the general physician or a medical specialist. SAEs need to be reported till the end of study within the Netherlands

### Frequency and plans for auditing trial conduct {23}

Data obtained during this study will be monitored annually by an independent monitor. The monitor will randomly check 10 participants to ensure the trial protocol is followed. An audit or inspection may take place during the study, performed by the sponsor and/or the regulatory authorities. These will check whether the study is conducted in accordance with legislation and the study protocol. The trial steering committee annually reports the progress of the study to the accredited medical ethics committee and will report the findings of the study at the end of the study. The accredited medical ethics committee will meet annually, after having received the annual study progress via a progress report of the trial steering committee, to review the conduct of the study.

### Plans for communicating important protocol amendments to relevant parties (e.g. trial participants, ethical committees) {25}

Any modifications to the study protocol, which may impact the conduct of the study or patient safety, including changes of the study objectives, study design, patient population, sample size, study procedures or significant administrative aspects, will be sent for approval to the Medical Ethics Committee of the Erasmus MC prior to implementation, and the health authorities will be informed in accordance with local regulations. Patients will be informed, if deemed appropriate, about amendments, and will be asked whether they want to continue their participation.

## Dissemination plans {31a}

On completion of the study, its findings will be published in peer-reviewed journals and presented at national and international scientific conferences to publicize the research to healthcare professionals, health services authorities and the public. A summary of the results will be made available to the study patients if requested.

## Discussion

This trial will assess the effectiveness of ICU-VR to improve psychological well-being and quality of life in patients diagnosed with COVID-19 warranting ICU treatment, as part of the post-COVID follow-up clinic. The results of this study will provide insight whether ICU-VR is a useful modality that can be implemented in the post-ICU follow-up initiatives as part of routine ICU aftercare.

Previous interventions to improve psychological sequelae and quality of life following ICU treatment include the use of ICU diaries, primary care follow-up programmes and ICU follow-up clinics. Unfortunately, none of these has conclusively demonstrated an improvement in quality of life [18, 20, 47, 48]. As such, there currently is no evidence-based intervention to improve psychological sequelae or quality of life after ICU treatment, and uniform guidelines for the organization of post-ICU care are largely lacking.

Exposure therapy using VR to treat non-ICU-related anxiety and PTSD has demonstrated to be equally effective as in vivo exposure, the gold standard for PTSD and anxiety treatment, and was preferred by patients [28, 49, 50]. Previously, we demonstrated that a sepsis specific ICU-VR intervention improved psychological recovery and mental quality of life in sepsis survivors after ICU treatment [33]. The ideal timing of ICU-VR remains however unknown and the question remains whether such a novel modality can be structurally implemented in routine ICU aftercare, such as in post-ICU follow-up clinics, and whether ICU-VR could be applied for recovery in other illnesses. Most ICU follow-up initiatives are organized 3 months after discharge; consequently, implementation at this time-point would result in a much more feasible implementation of the intervention in existing ICU aftercare programmes [51]. We therefore wanted to determine its effect 3 months after hospital discharge. In a previous study, ICU-VR was applied in a very early stadium, i.e. 8 days after ICU discharge, which could limit its implementation because this would increase work-load [33]. Because the burden for critical care services has risen exponentially in response to the COVID-19 pandemic, we were especially interested to assess its effect as part of our regional ICU aftercare and its effect in COVID-19 patients. Additionally, it is unknown whether a later intervention could also relieve stress and improve quality of life. We therefore also studied the effect of ICU-VR exposure 6 months post-discharge in the control group.

### Limitations

There are some limitations to our study design. First, as our previous research has only revealed the effect of ICU-VR offered in the first weeks after ICU discharge, we had to assume a comparable effect size when delivered 3 months after discharge. This may increase the risk of a type II error. To minimize this risk, we decided to include in pre-specified time period, and inclusion will be continued in case our enrolment aim is reached within this period. Furthermore, as the outcome variables were not normally distributed in our previous study, we did not assume any distribution, which may have resulted in an overestimation of our required sample size. Second, the generalization of results from this study may be limited because patients were only included at four different hospitals in a confined area in the Netherlands. Third, as we believe that the effect of our intervention is partially dependent on exposure to the actual environment where a patient was treated, we used hospital-specific ICU-VR, limiting easy implementation in other hospitals. Last, due to the nature of the intervention, neither investigators nor participants could be blinded to patients’ allocation. To reduce this bias, analysation will be conducted blind.

## Conclusions

We designed this multicentre, randomized clinical trial to evaluate the effect of an intensive care unit-specific virtual reality intervention, offered 3 months after hospital discharge, on the psychological well-being and quality of life in patients diagnosed with COVID-19 warranting ICU treatment. Results from this trial will provide insight whether virtual reality is a modality that can be implemented to improve ICU aftercare and can be adapted for specific diseases.

## Trial status

The initial version of the study protocol (version 1.2) was approved on June 10, 2020. The current version of the study protocol (version 1.3) was approved on August 26, 2020. The study’s data collection is currently ongoing, recruitment of participants has started June 29, 2020, and has ended February 3, 2021.

## Supplementary Information

**Additional file 1.** Film script of the COVID-19 intensive care unit-specific virtual reality intervention.

## Abbreviations

COVID-19: Coronavirus disease 2019
HADS: Hospital Anxiety and Depression Scale
HMD-VR: Head-mounted display virtual reality
HRQoL: Health-related quality of life
ICU: Intensive care unit
ICU-VR: Intensive care unit-specific virtual reality
IES-R: Impact of Event Scale–Revised
PCR: Polymerase chain reaction
PICS: Post-intensive care syndrome
PTSD: Post-traumatic stress disorder
RAND-36: Research and development 36-items
SARS-CoV-2: Severe acute respiratory syndrome coronavirus 2
VR: Virtual reality
