# Supplementary material for: Effect of intensive care unit-specific virtual reality (ICU-VR) to improve psychological well-being and quality of life in COVID-19 ICU survivors: a study protocol for a multicentre, randomized controlled trial
Source: Trials. 2021 May 5;22:328. doi: 10.1186/s13063-021-05271-z (PMC8097671; doi:10.1186/s13063-021-05271-z)
Supplement: Supplementary file 1 — Additional file 1. Film script of the COVID-19 intensive care unit-specific virtual reality intervention. [file 13063_2021_5271_MOESM1_ESM.pdf]

Additional file 1.

Film script of the COVID-19 intensive care unit-specific virtual reality intervention.

Johan H. Vlake, BSc; Jasper Van Bommel, MD, PhD; Evert-Jan Wils, MD, PhD, et al. **Study protocol for a multicentre, randomised controlled trial evaluating the effect of intensive care unit-specific virtual reality (ICU-VR) on psychological distress and quality of life in COVID-19 ICU survivors.**

Copyright © 2020. Erasmus MC, Rotterdam, the Netherlands. All rights reserved. For use: contact m.vangenderen@erasmusmc.nl.

Scene 1. Introduction by an ICU physician and a nurse.

Setting: The ICU physician and nurse are placed in front of the ICU.

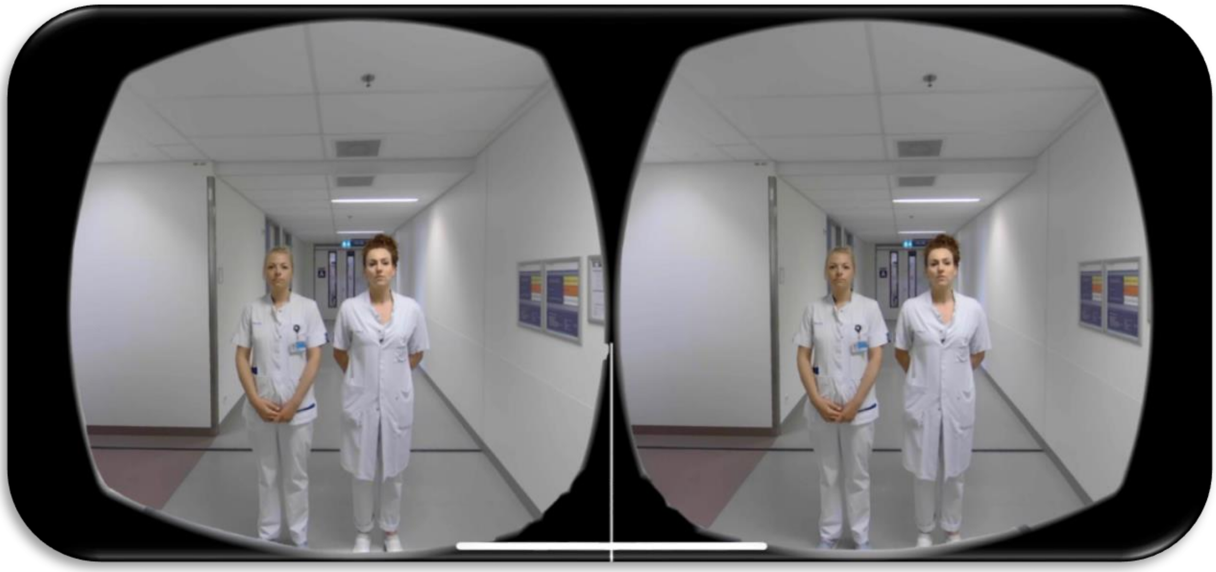

**ICU physician:**	Hello, welcome to this virtual environment. My name is **'name physician'**, one of the physicians in this ICU.
**ICU nurse:**	Hello, I am **'name nurse'**, one of the nurses in this ICU.
**ICU physician:**	You were treated in the ICU for the new coronavirus. In this virtual environment, we provide you with explanations about the ICU and about the treatment you received here.
**ICU nurse:**	We will join you during this experience, but we will first lay you down on an ICU bed.

Setting: The patient will be virtually installed on an ICU bed during a fade in-fade out.

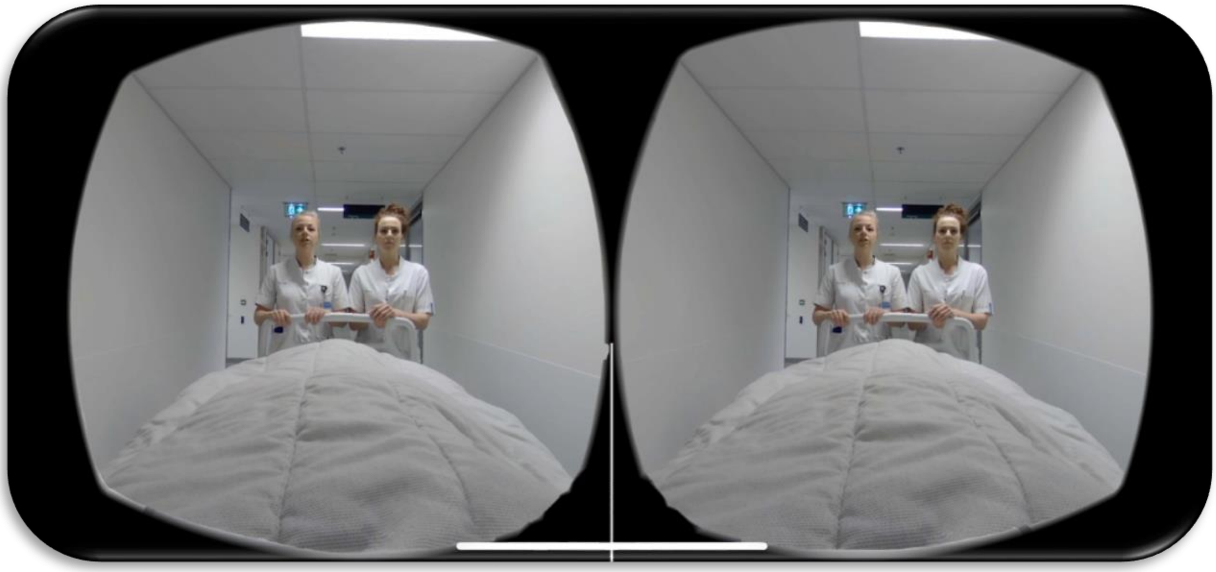

**ICU nurse:**	We will now bring you to your ICU room.

Setting: The ICU physician and ICU nurse will bring the patient to one of the ICU rooms while walking over the intensive care department.

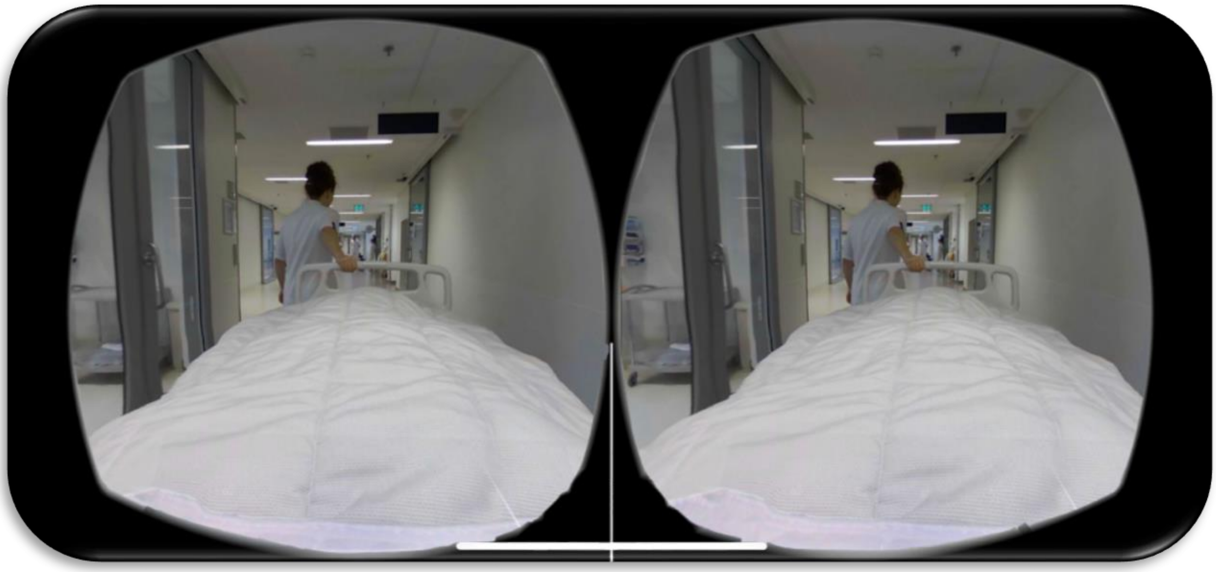

Voice-over: Intensive care means intensive and special care for critically ill patients, where the most important vital functions, such as the respiratory rate, oxygen saturation and heart rate, can be monitored and supported, if needed. Therefore, this department is different from other departments. The intensive care department consists of several one-patient ICU rooms and a post for nurses located in the middle of the department. In an ICU room, circumstances and materials are available to offer critically ill patients the optimal treatment. Moreover, the chances of hospital acquired infections and medication failures are minimal, and a quiet environment is provided. If you look around, you'll see the intensive care department. At the nurse post, nurses are present throughout the day, as are monitors. Nurses can also monitor patients physically through the windows of the room, which allows nurses to be able to continuously keep an eye on you.

Setting: The patient arrives at the ICU room, and the ICU physician and ICU nurse place the patient on the bed in the ICU room.

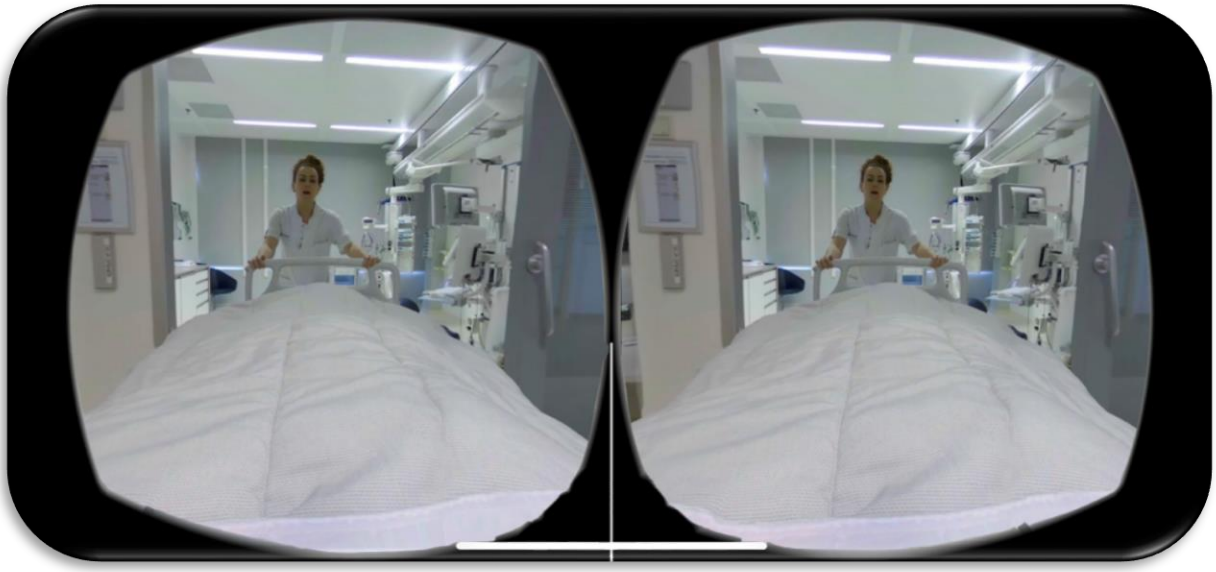

ICU physician: We are now entering an ICU room. Here, you'll receive an explanation about intensive care treatment. We will first explain the devices in the room, which are placed next to you. We will now leave the room and will come back after the explanation.

Setting: The ICU physician and ICU nurse will leave the room.

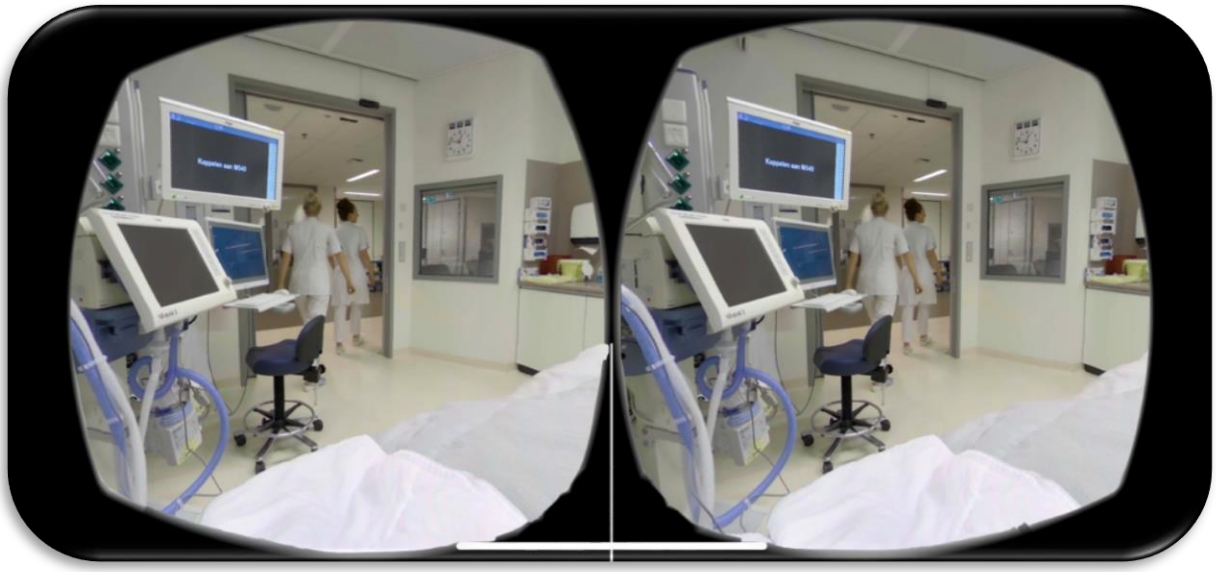

Scene 2. Explanation of the devices and alarm noises.

Voice-over: There are several devices next to you, such as a monitor, medication pumps and a mechanical ventilator; look around you. These devices are needed to monitor you. Each device has its own functions and alarm noise. We will now explain these to you.

Setting: The surveillance monitor is outlined.

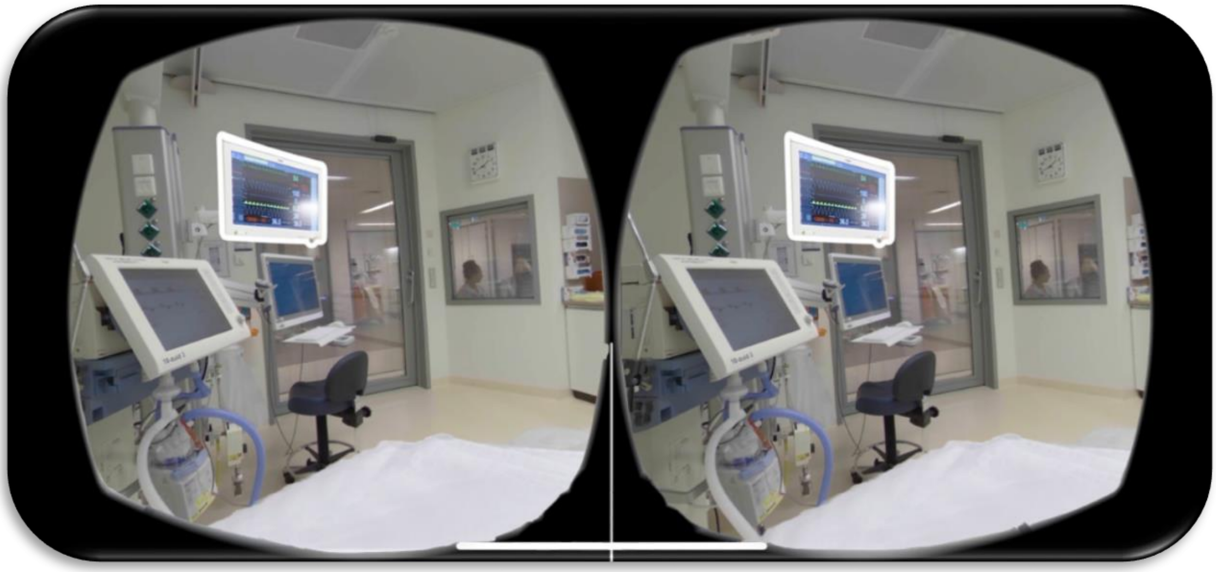

Voice-over: When you look to your left, you'll see the surveillance monitor.

Setting: A white arrow appears that points from the surveillance monitor to an explanation window in front of the patient, where the surveillance monitor is animated.

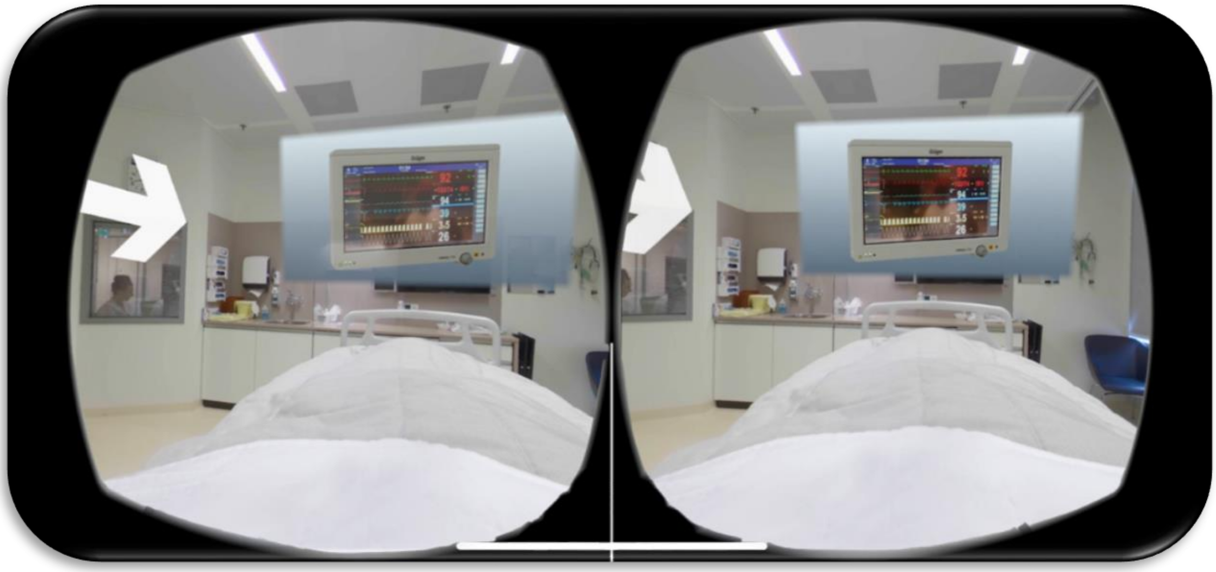

Voice-over: When you look forward again, we will explain the function of the surveillance monitor. The surveillance monitor monitors heart rate, blood pressure, respiratory rate, and oxygen saturation. If, for instance, your blood pressure is too low, the following alarm signal is produced.

ALARM SIGNAL SURVEILLANCE MONITOR>

Setting: The explanation window in front of the patient disappears. The medication pumps are outlined.

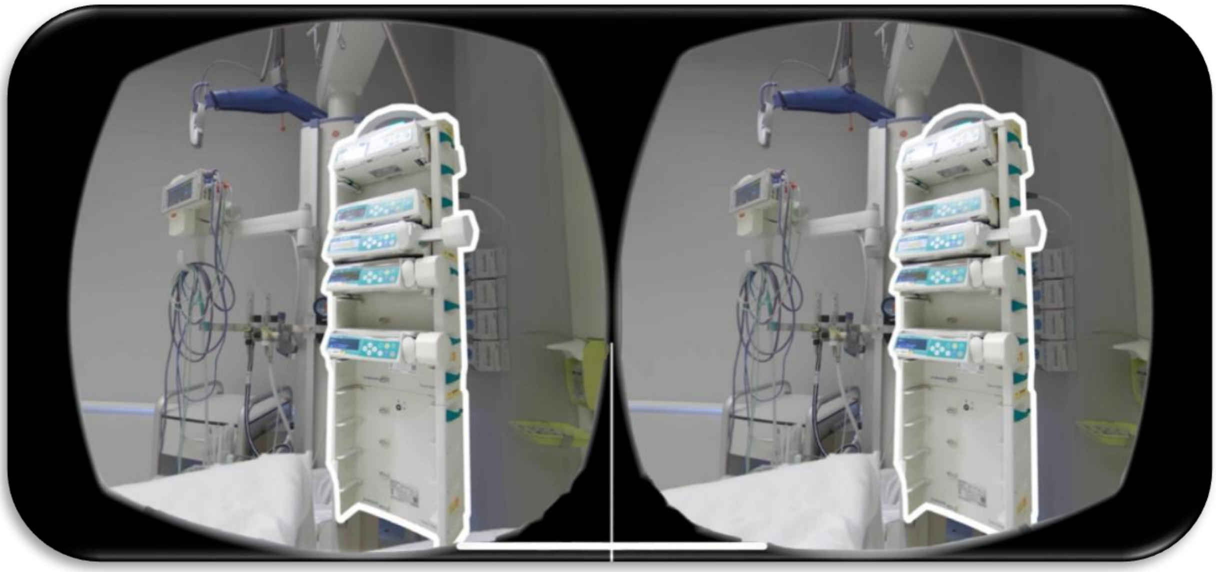

Voice-over: If you look to your right, you'll see the medication pumps.

Setting: A white arrow appears that points from the medication pumps to an explanation window in front of the patient, where the medication pumps are animated.

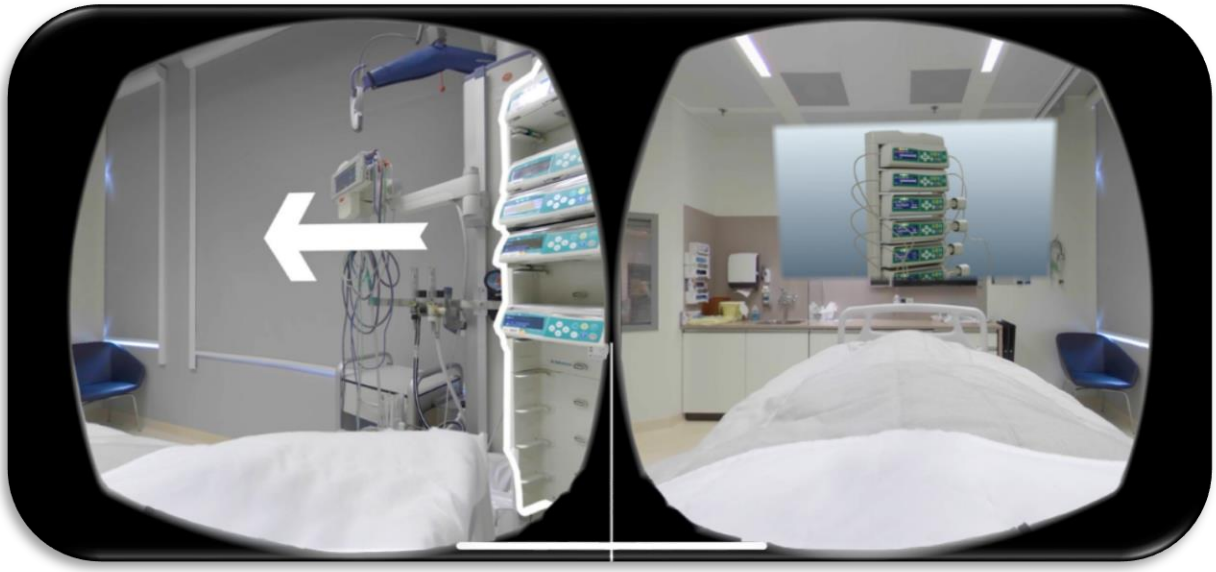

Voice-over: These pumps are used to give medication. When you hear the following sound,

<ALARM SIGNAL MEDICATION PUMPS>

the nurse is warned that your medication is almost empty.

Setting: The explanation about medication pumps disappears, and an animation appears in the explanation window explaining intubation and mechanical ventilation.

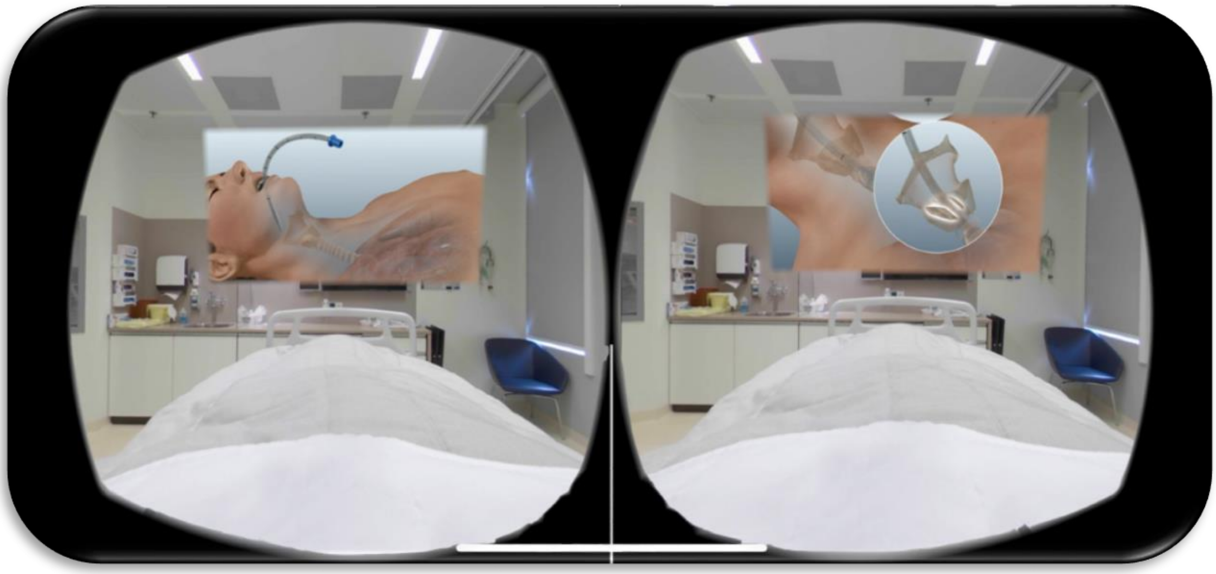

Voice-over: Because you were critically ill, we decided to support your breathing. This was done to maintain the appropriate amount of oxygen in your body. To support your breathing, we inserted a tracheal tube through your mouth into your trachea. Because this procedure is often uncomfortable, you were sedated during the insertion of the tube. At the end of the tube, there is a small air balloon, which is filled with air. This balloon prevents the leakage of oxygen and the contents of the stomach from entering the lungs. Due to the placement of the tube between the vocal cords, patients cannot talk when they are intubated. When the lungs have sufficiently recovered, the tracheal tube can be removed. The tracheal tube is frequently cleaned by suctioning the tube. Hereby, mucus will be removed to prevent infections. Sometimes, it will be enough to do this once, but this has to be repeated often.

Setting: The explanation window disappears. The mechanical ventilator is outlined.

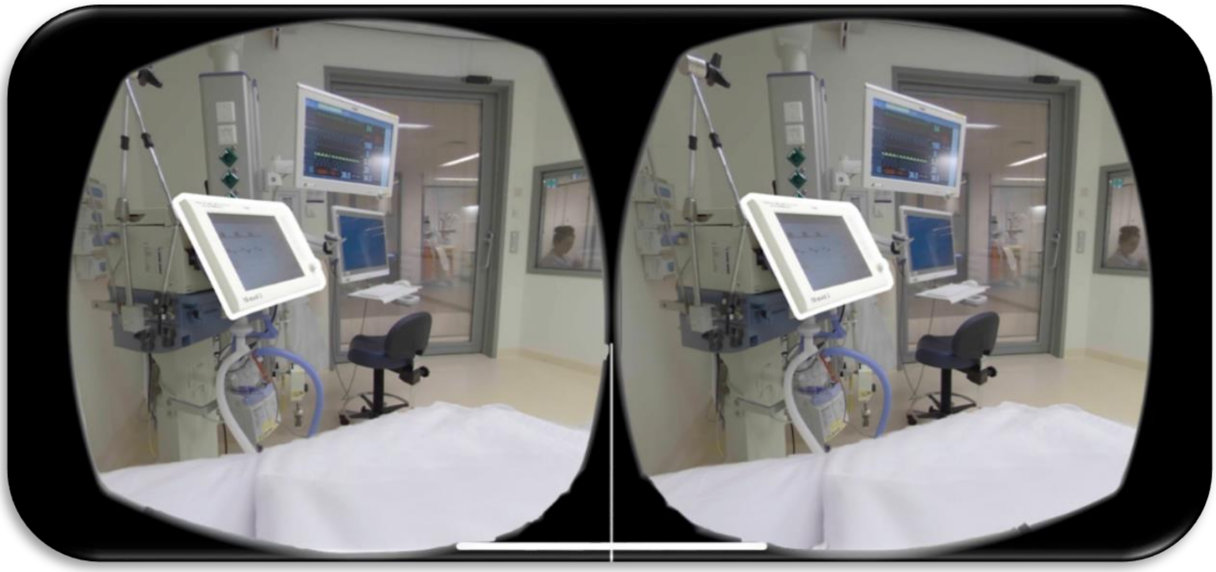

Voice-over: If you look to your left, you'll see the mechanical ventilator.

Setting: A white arrow appears that points from the mechanical ventilator to an explanation window in front of the patient, where the mechanical ventilated is animated.

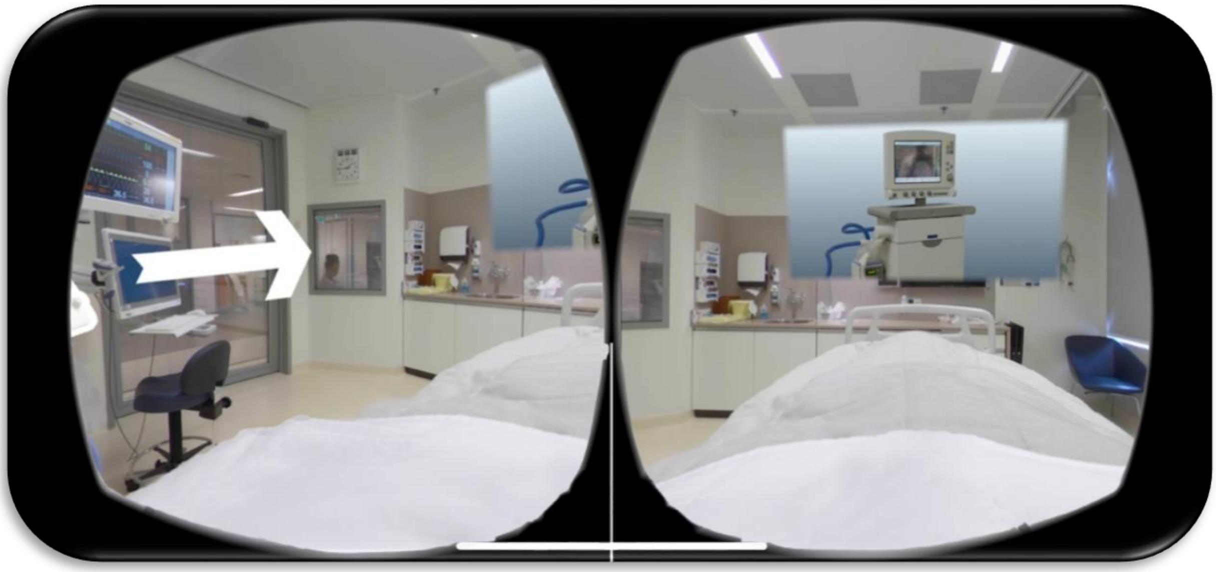

Voice-over: When you look in front of you, we will give you a further explanation about the mechanical ventilator. The mechanical ventilator supports your breathing. If you hear the following sound,

<ALARM SIGNAL MECHANICAL VENTILATOR>

the nurse is warned.

Setting: The animation of the mechanical ventilator disappears, and the explanation about prone positioning is animated in the explanation window.

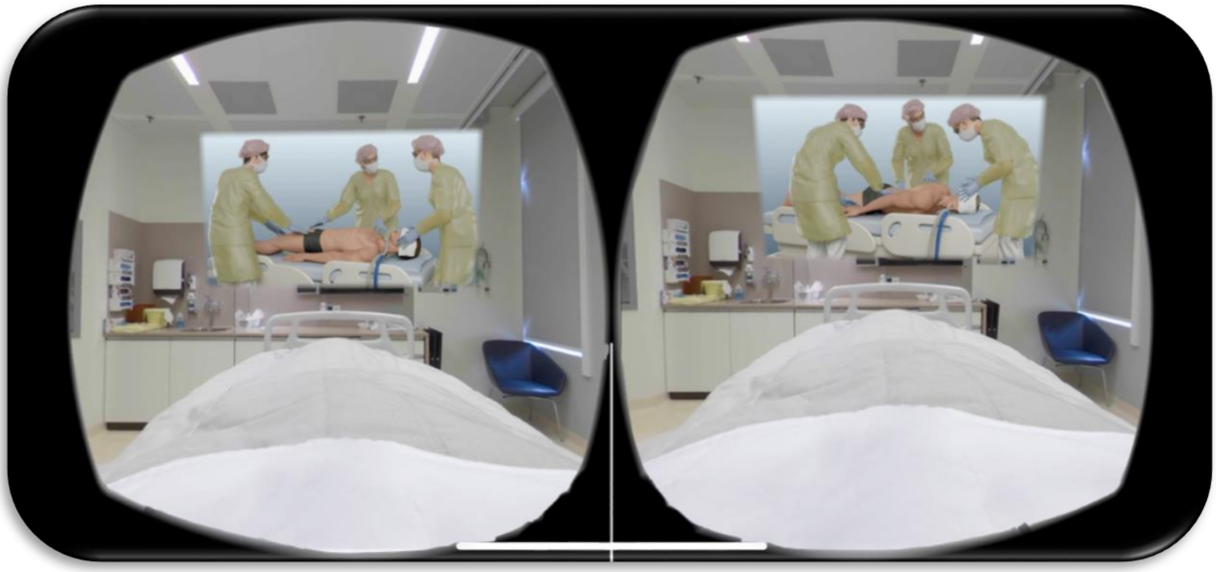

Voice-over: As a consequence of several diseases, including coronavirus, the alveoli and pulmonary vessels can partially close, resulting in the body being unable to absorb sufficient oxygen. There are relatively more alveoli in the back of the lungs. In the occasion mechanical ventilation in a normal position is no longer effective, it can be decided to ventilate patients in the prone position or laying

on their stomach. The alveoli and pulmonary vessels in the back of the lungs are thereby better ventilated, hopefully resulting in better absorption of oxygen. Often, there is an immediate improvement in the mechanical ventilation conditions after prone positioning. To prevent pressure marks on the face, the eyes are protected and the head is placed in a position to the side. Over time, the positive effect of this prone position diminishes, and the patient is again placed on their back. Therefore, it is often decided to ventilate in prone positioning for several hours and thereafter again on the back for several hours. Because prone positioning can be uncomfortable, patients are sedated.

Scene 3. Explanation concerning the drips, infusions and gastric tube.

Setting: The explanation window disappears, and the ICU physician appears.

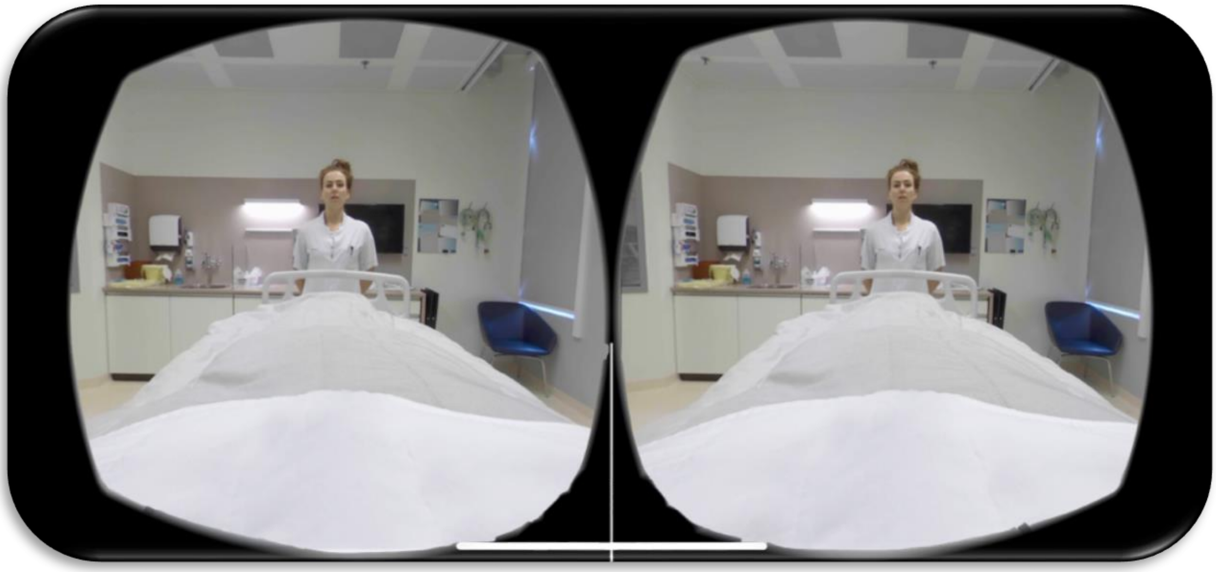

ICU physician: The different devices, the mechanical ventilator and the alarm signals have just been explained to you. Now, you will receive an explanation concerning the drips, infusions and gastric tube.

Setting: The ICU physician disappears.

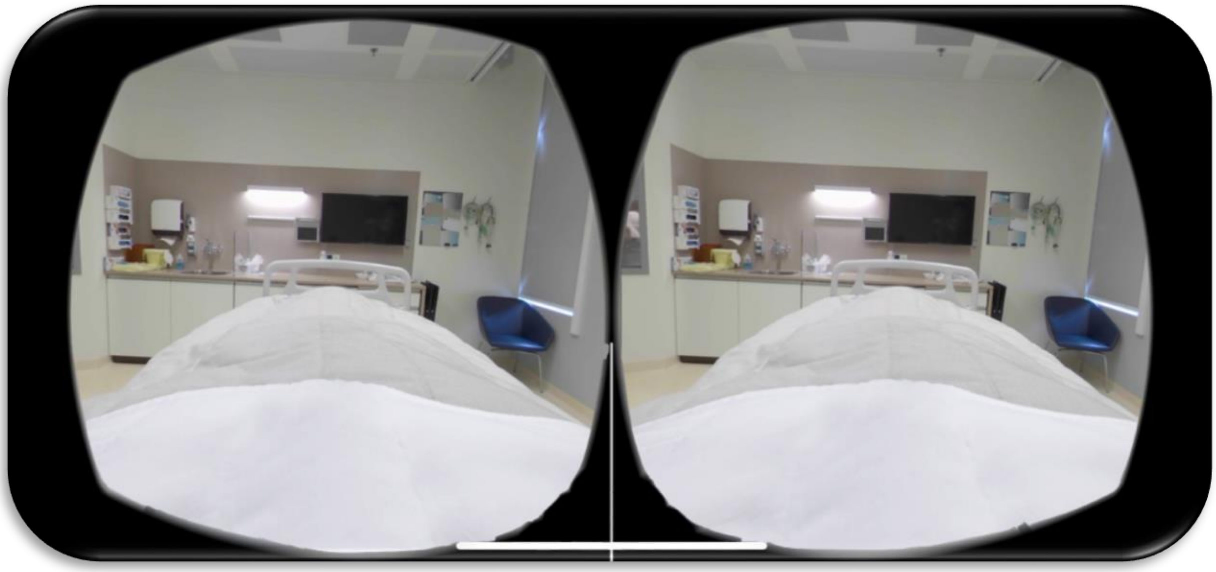

Voice-over: IV drips and lines are necessary not only to administer medication and fluids but also to continuously monitor the blood pressure.

Setting: The explanation window appears, and the function of a peripheral drip is explained using an animation.

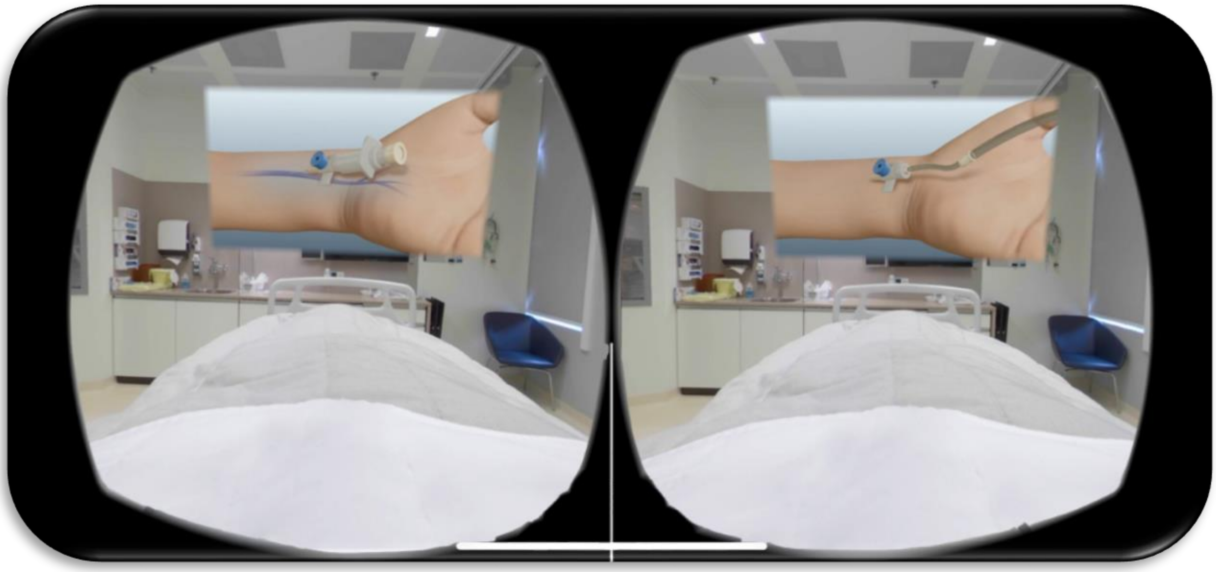

Voice-over: This is an ‘ordinary’ IV drip, also called a peripheral IV drip. This is usually inserted into a vessel in the forearm, but sometimes, it is placed in the foot. The nurse can administer medication or fluid through this drip. Because these peripheral vessels are thin, not every medication can be administered through the veins.

Setting: Explanation of a central line is explained using an animation.

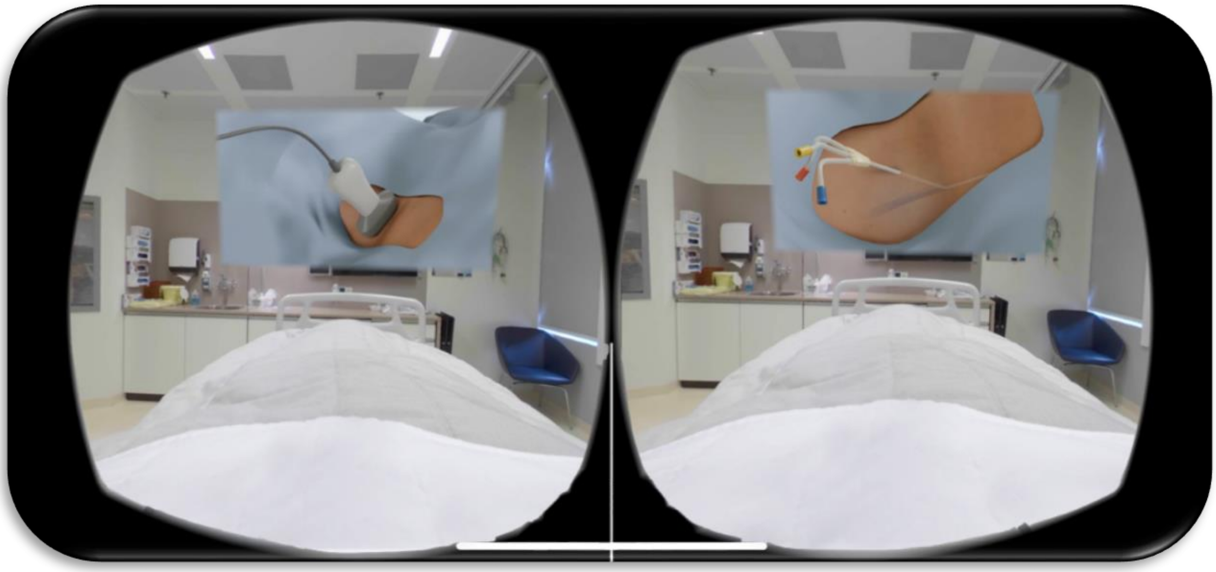

Voice-over: Here, you see a central line. This is a think IV drip that is inserted into a large blood vessel, often in the neck or groin. The insertion of such a line will be performed in a sterile manner; therefore, a blue cloth is stretched over your head. Working in a sterile field minimises the risk of infection. The main reason to insert a central line is to administer medications that cannot be administered through ordinary IV drips. Nutrition can also be directly administered to the blood stream through a central line.

Setting: Explanation of an arterial line is explained using an animation.

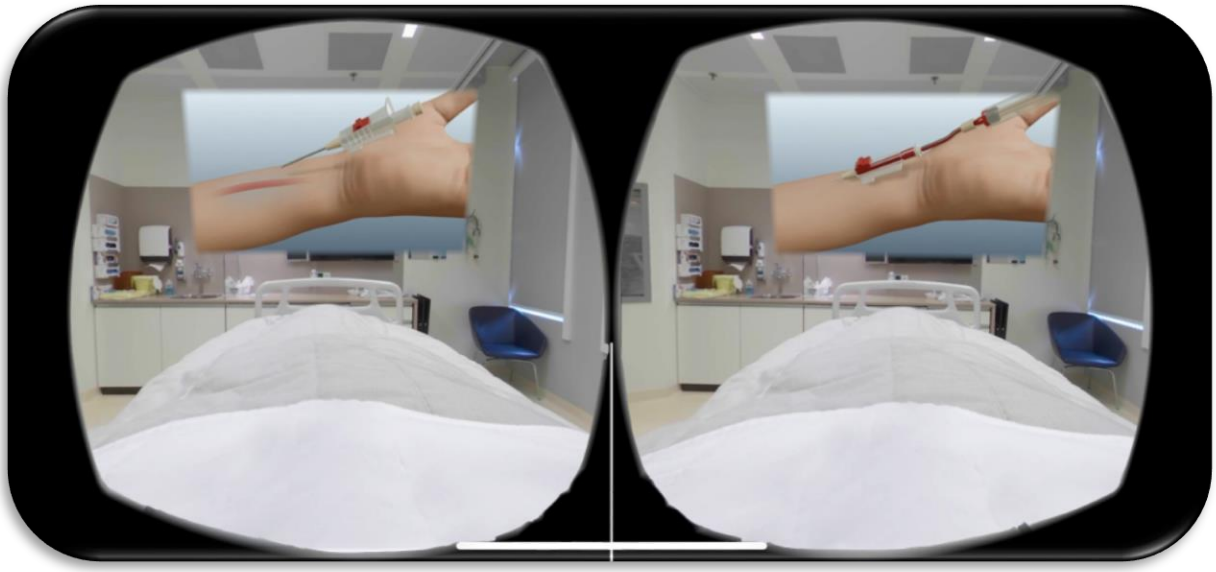

Voice-over: This is an arterial line. This is an IV drip that is placed directly into an artery, so blood pressure can continuously be monitored. It is also used to take blood samples. Without such a line, blood samples may have to be taken too often.

Setting: Explanation about a gastric tube is given using an animation.

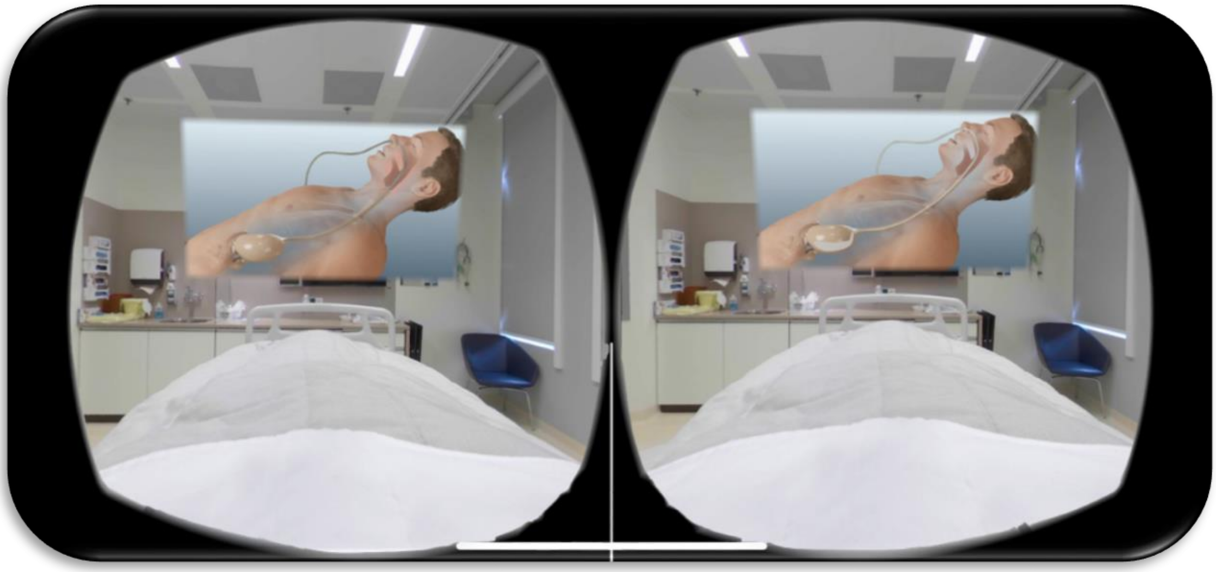

Voice-over: A gastric tube is a tube that is placed through the nose or mouth through the oesophagus into the stomach. The tube is usually to administer tube feedings. It can also be used to administer medications.

Setting: The tracheotomy procedure is explained using an animation.

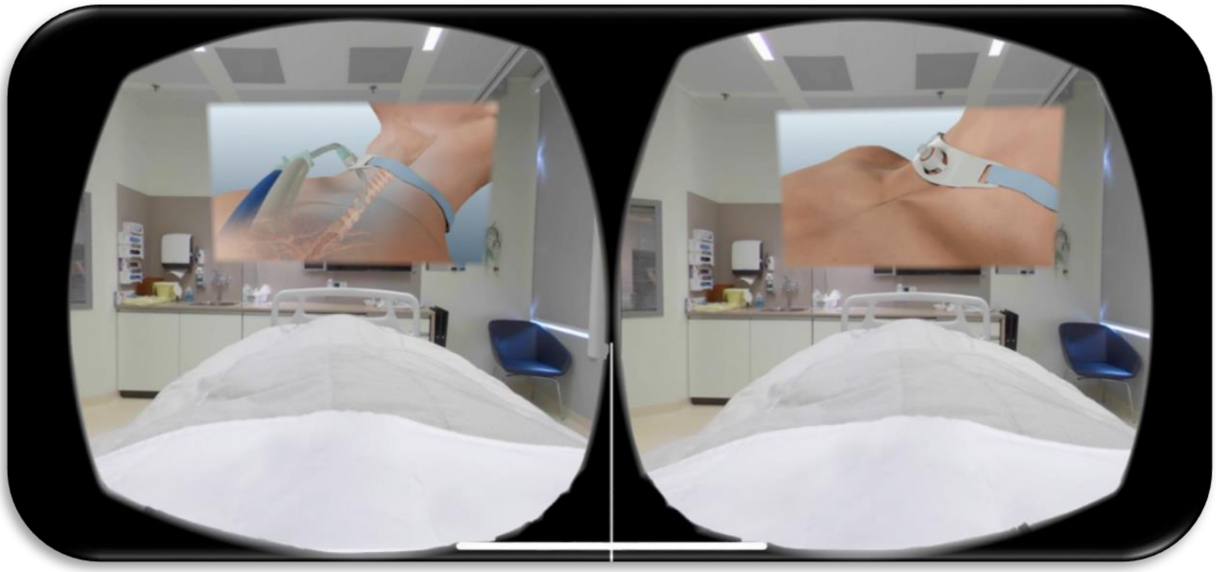

Voice-over: When patients are mechanically ventilated for a prolonged period of time, they sometimes receive a tracheotomy. During a tracheotomy procedure, a tube (also known as a cannula) is placed in the trachea through the neck. This cannula replaces the ventilation tube, which is inserted through the mouth. There are several reasons to perform a tracheotomy, but the most important one is long-term mechanical ventilation. The patient must be slowly and gradually weaned off mechanical ventilation. Tracheotomy placement is often conducted in the ICU. The cannula is inserted just above the sternum through an incision in the trachea. The end of the tube can be inflated to prevent air leakage. Because the air flows through the cannula to the lungs and no air passes the vocal cords, patients initially cannot speak when they have a tracheotomy. However, the tracheal cannula can be closed using a speaking valve, whereby the end of the cannula is deflated; as a result, air will flow through the vocal cords making it possible to speak. The tracheostomy will be removed when a patient has sufficient strength to breath on their own and can cough up sputum properly.

Scene 4. Explanation about the treatment team and their responsibilities.
Setting: The explanation window disappears, and an ICU physician, nurse and resident enter the room.

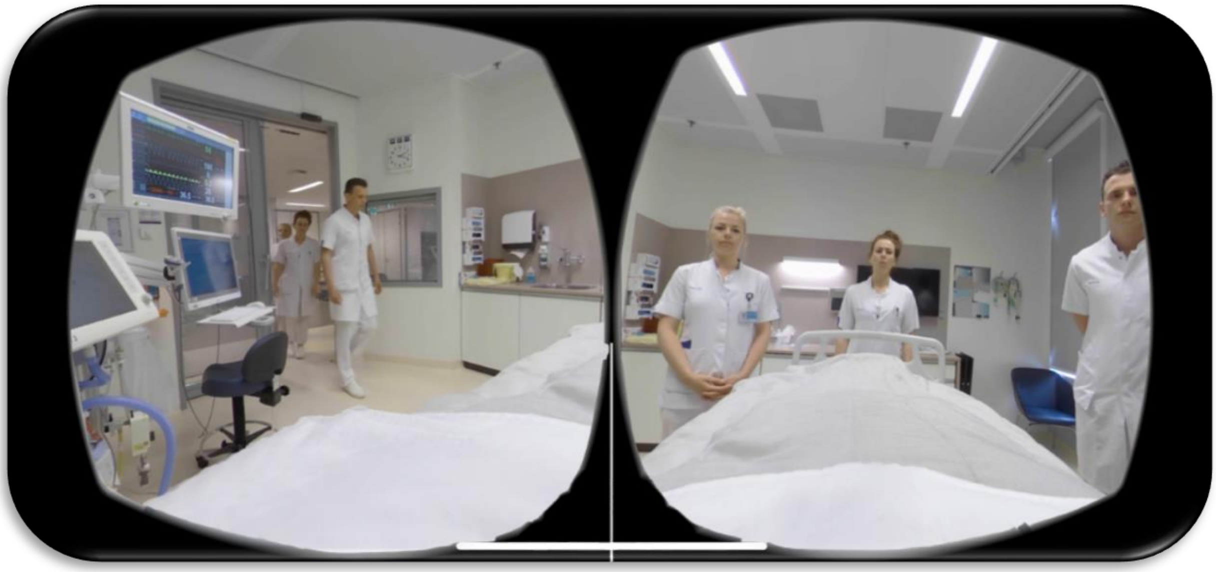

**Voice-over:**	In the ICU, you are treated 24 hours per day by a treatment team. Therefore, there are many people working in the ICU.<br>The medical treatment team that is primarily responsible for your treatment includes the ICU physician, the ICU resident and the ICU nurse.
**ICU physician:**	My fellow ICU physicians and I, the intensivists, are specialised in the treatment of critically ill patients. Every morning, afternoon and evening, there is a meeting with the treatment team taking care of you to discuss how you are doing. This will take place in your room.
**ICU nurse:**	My fellow ICU nurses and I will look after you, monitor you continuously and are trained to operate the devices for your treatment. You will be taken care of by the same nurse every shift.

Setting: The treatment team leaves the room.

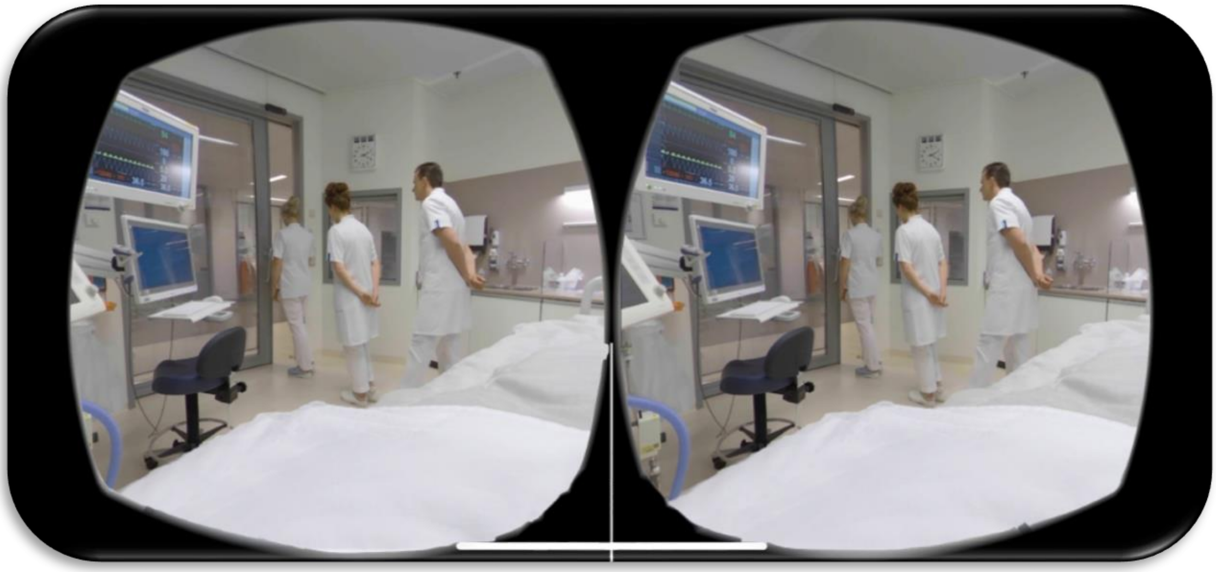

Scene 5. Explanation about isolation and personal protection measures.

Voice-over: During your stay in the ICU, you are treated in isolation. Isolation measures are aimed at preventing the spread of microorganisms, such as coronavirus. These measures are in addition to the basic hygiene measures. We will now show you how this was done.

Setting: The treatment team returns to the room with isolation measures.

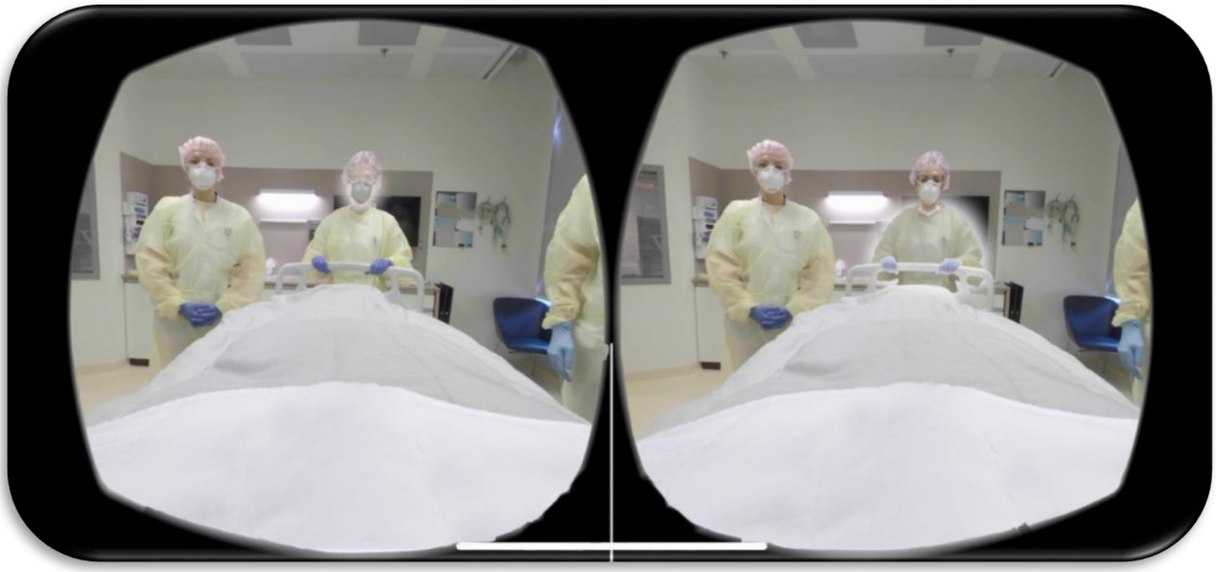

Voice-over: The treatment team has applied isolation measures when entering the room by wearing personal protective equipment. Before entering the room, the team was therefore wearing: Non-sterile gloves, a mouth-nose mask, an isolation apron with long sleeves, safety glasses, a hair cap.
Prior to leaving the room, the personal protective equipment is removed and hands are disinfected.

Scene 6. Explanation about SARS-CoV-2 and COVID-19.

Setting: The treatment team leaves the room. An explanation window appears to give information about the coronavirus.

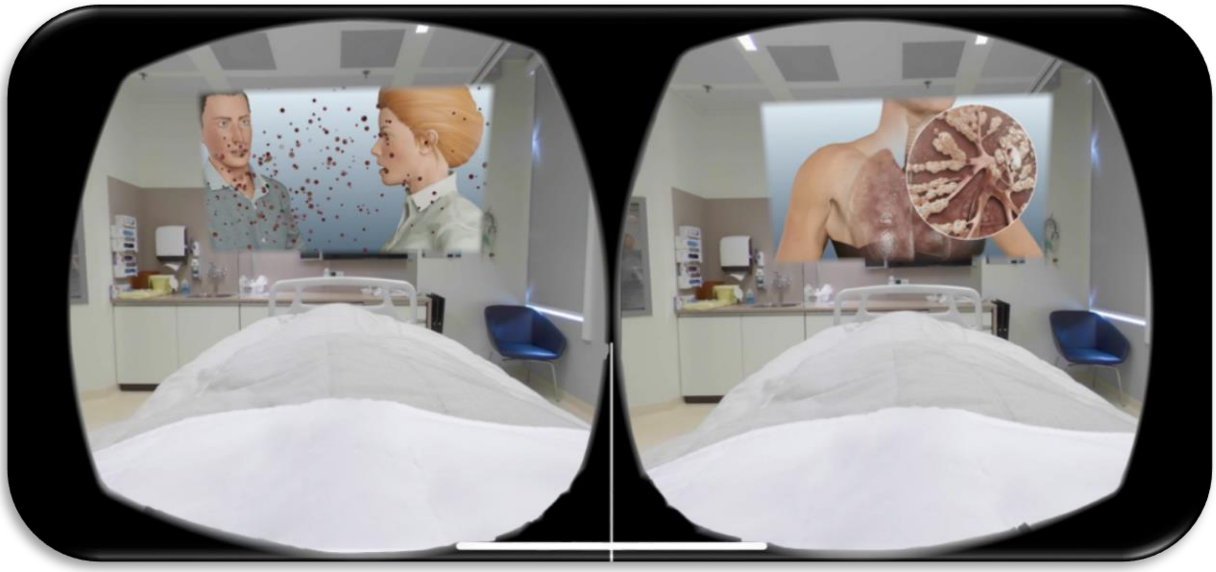

Voice-over: You were treated in the ICU due to an infection with the new coronavirus, SARS-CoV-2, causing COVID-19. This virus was discovered at the end of 2019 in China and rapidly spread around the world.

You were infected because you have had contact with a person who was also infected by the virus, for instance, because this person sneezed. The virus can enter the body through the mouth, nose and eyes.

When the coronavirus enters the body, you can develop several symptoms. These often resemble a cold and often manifests as complaints of fever, increased heart rate and/or coughing. Coronavirus can cause pneumonia. This happens when the virus enters the alveoli and cells of the lung, reducing the capacity to absorb oxygen.

In some individuals, the disease can cause severe pneumonia, requiring temporary support. In your care, you were supported in the ICU during your COVID-19 illness. There is still no cure available for the new coronavirus, and the body must therefore clear the virus by itself. When the body recovered sufficiently, you were discharged from the ICU.

Scene 7. Outro

Setting: The explanation window disappears and the ICU physician and nurse re-enter the room.

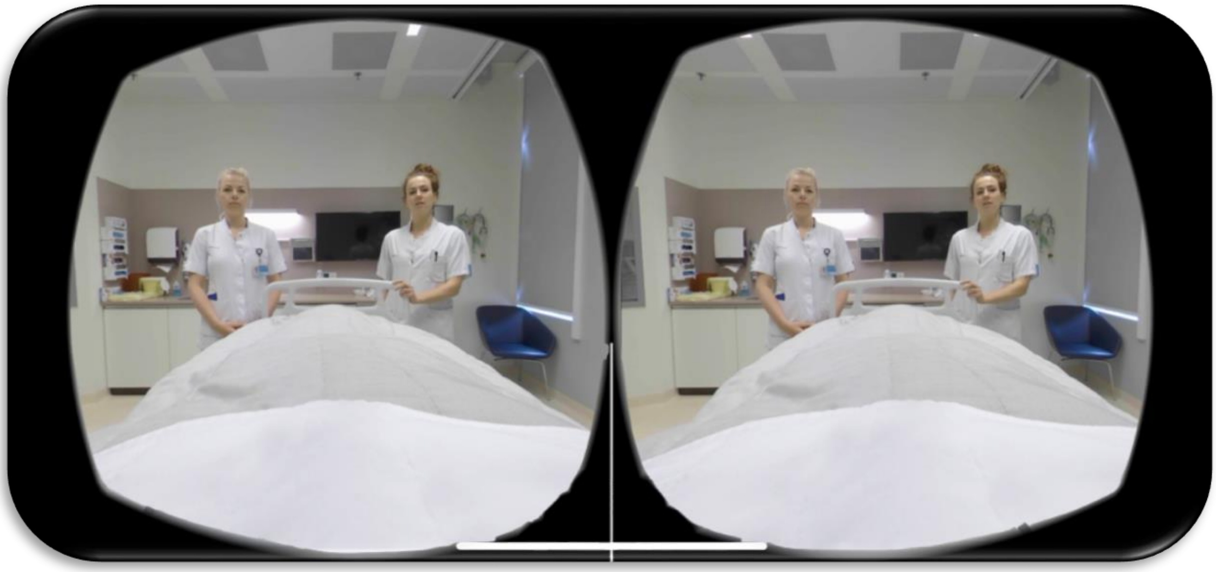

ICU physician: We hope you now have a better understanding of the treatment you received in the ICU. This is the end of this video, you can remove the VR glasses.
